# *In vitro* Antiplasmodial and Molecular Docking Studies of Chemical Constituent Isolated from the Bark of *Diospyros lanceifolia* (Ebenaceae)

**DOI:** 10.21315/tlsr2025.36.2.10

**Published:** 2025-07-31

**Authors:** Ibrahim Dankane Bafarawa, Muhammad Solehin Abd Ghani, Arba Pramundita Ramadani, Shofiatul Fuadah, Sista Werdyani, Unang Supratman, Muhammad Bisyrul Hafi Othman, Mohamad Nasir Mohamad Ibrahim, Khalijah Awang, Marc Litaudon, Mohammad Tasyriq Che Omar, Habibah A. Wahab, Mohamad Nurul Azmi

**Affiliations:** 1 School of Chemical Sciences, Universiti Sains Malaysia, 11800 USM, Pulau Pinang, Malaysia; 2 Umaru Ali Shinkafi Polytechnic Sokoto, P. M. B. 2356 Sokoto State, Nigeria; 3 Department of Pharmacy, Universitas Islam Indonesia, Jl. Kaliurang KM 14.4 Sleman, Yogyakarta 55584, Indonesia; 4 Department of Chemistry, Faculty of Mathematics and Natural Sciences, Universitas Padjadjaran, 45363 Jatinangor, Indonesia; 5 Department of Chemistry, Faculty of Science, Universiti Malaya, 50603 Kuala Lumpur, Malaysia; 6 Institute de Chimie des Substances Naturelles, CNRS-ICSN UPR2301, Univ. Paris-Sud 11, av. de la Terrasse, 91198 Gif-sur-Yvette, France; 7 Biological Section, School of Distance Education, Universiti Sains Malaysia, 11800 USM, Pulau Pinang, Malaysia; 8 School of Pharmaceutical Science, Universiti Sains Malaysia, 11800 USM, Pulau Pinang, Malaysia

**Keywords:** *Diospyros lanceifolia*, *Plasmodium falciparum* FCR3, Antiplasmodial Activity, Molecular Docking, *Pf*ATP6 Protein, *Diospyros lanceifolia*, *Plasmodium falciparum* FCR3, Aktiviti Antiplasmodial, Penyambungan Molekul, Protein *Pf*ATP6

## Abstract

The phytochemical investigations of the ethyl acetate bark extract of *Diospyros lanceifolia* have led to the isolation of eight compounds, namely lupeol (**1**), betulin (**2**), β-sitosterol (**3**), oleic acid (**4**), α-amyrin acetate (**5**), glyceryl trilinoleate (**6**), β-amyrin (**7**) and shinanolone (**8**). The structures of all compounds were established using various spectroscopic techniques such as 1D and 2D-NMR, FT-IR and HRESIMS, which were then compared with reported literature for validation. All compounds isolated from this plant were screened for an *in vitro* study against *Plasmodium falciparum* FCR3 followed by an *in silico* molecular docking study with the *Pf*ATP6 protein. The in vitro results revealed that five compounds exhibited strong to good activity (IC_50_ < 10 μM). In order of potency, these compounds include **5, 3, 6, 1** and **4** with IC_50_ values of 0.3 ± 0.3 μM, 0.3 ± 0.3 μM, 1.9 ± 2.2 μM, 4.4 ± 7.4 μM and 8.4 ± 4.9 μM, respectively. Compounds **5** and **3** showed the strongest activity compared to the control drugs artemisinin and chloroquine, with the IC_50_ of 0.7 ± 0.3 μM and 10.3 ± 2.9 μM, respectively. The *in silico* molecular docking simulations showed that all active compounds from the *in vitro* study displayed good binding affinity to the *Pf*ATP6 protein binding site, with compounds **3, 1** and **5** demonstrating greater binding affinity compared to the other compounds tested, including artemisinin and chloroquine. All compounds exhibited several hydrophobic interaction modes with amino acids of *Pf*ATP6 residues. Interestingly, all compounds exhibited hydrogen bonding with ASN1039 residue, except compound **3**. The *in silico* study of these compounds supports the *in vitro* antiplasmodial activity findings, suggesting that these compounds are potential lead candidates for the development of new antiplasmodial drugs.

## Abstrak

Siasatan fitokimia terhadap ekstrak kulit etil asetat bagi *Diospyros lanceifolia* telah membawa kepada pengasingan lapan sebatian, iaitu lupeol (**1**), betulin (**2**), β-sitosterol (**3**), asid oleik (**4**), asetat α-amyrin (**5**), trigliserida trilinoleat (**6**), β-amyrin (**7**) dan shinanolone (**8**). Struktur semua sebatian telah ditentukan menggunakan pelbagai teknik spektroskopi seperti 1D dan 2D-NMR, FT-IR dan HRESIMS, yang kemudiannya dibandingkan dengan literatur yang dilaporkan untuk pengesahan. Semua sebatian yang diasingkan dari tumbuhan ini telah disaring untuk kajian *in vitro* terhadap *Plasmodium falciparum* FCR3 diikuti dengan kajian pengikatan molekul *in silico* dengan protein *Pf*ATP6. Hasil *in vitro* menunjukkan bahawa lima sebatian menunjukkan aktiviti yang kuat hingga baik (IC_50_ < 10 μM). Mengikut urutan potensi, sebatian-sebatian ini termasuk **5, 3, 6, 1** dan **4** dengan nilai IC_50_ masing-masing 0.3 ± 0.3 μM, 0.3 ± 0.3 μM, 1.9 ± 2.2 μM, 4.4 ± 7.4 μM dan 8.4 ± 4.9 μM. Sebatian **5** dan **3** menunjukkan aktiviti paling kuat berbanding dengan ubat kawalan artemisinin dan chloroquine, dengan IC_50_ masing-masing sebanyak 0.7 ± 0.3 μM dan 10.3 ± 2.9 μM. Simulasi pengikatan molekul *in silico* menunjukkan bahawa semua sebatian aktif dari kajian *in vitro* menunjukkan daya ikatan yang baik kepada tapak pengikatan protein *Pf*ATP6, dengan sebatian **3, 1** dan **5** menunjukkan daya ikatan yang lebih kuat berbanding dengan sebatian lain yang diuji, termasuk artemisinin dan chloroquine. Semua sebatian menunjukkan beberapa mod interaksi hidrofobik dengan asid amino residu *Pf*ATP6. Menariknya, semua sebatian menunjukkan ikatan hidrogen dengan residu ASN1039, kecuali sebatian **3**. Kajian *in silico* bagi sebatian-sebatian ini menyokong penemuan aktiviti antiplasmodial *in vitro*, mencadangkan bahawa sebatian-sebatian ini adalah calon utama yang berpotensi untuk pembangunan ubat antiplasmodial baru.

**Highlights**

- The phytochemical investigations of the ethyl acetate bark extract of Diospyros lanceifolia (Ebenaceae) led to the isolation of eight compounds.
- The *in vitro* antiplasmodial activity result showed that five compounds in order of potency (**5, 3, 6, 1** and **4**) demonstrated good activity with IC_50_ values ranging from 0.3 μM–8.4 μM.
- All potent compounds for the *in silico* molecular docking study were able to stably bind to *Pf*ATP6 protein residues via hydrogen bonds and multiple hydrophobic interactions.
- This is the first report on the antimalarial activity of *Diospyros lanceifolia* and its isolated compounds

## INTRODUCTION

Malaria, a blood-borne infectious disease, continues to pose a significant threat to both children and adults, particularly in tropical and subtropical regions of the world (Shibeshi *et al.* 2020). According to the World Malaria Report 2024 by World Health Organization (WHO), approximately 263 million cases of malaria were reported in 83 countries where the disease is endemic. Of these cases, 247 million which represent about 94% of all global cases, occurred in the WHO African region and resulted in more than 567,000 deaths (WHO 2024). *Plasmodium falciparum* is the most dangerous species among the *Plasmodium* parasite because it causes higher rates of mortality and morbidity which have a likelihood of drug resistance (Chaniad *et al.* 2021). Malaria is primarily transmitted to humans through the bites of female *Anopheles* mosquitoes carrying the disease, in which the *Plasmodium* parasites penetrate the liver of humans to mature and reproduce in the bloodstream leading to malaria infection (Pan *et al.* 2018). However, the development of drug resistance of the malaria parasite to the first line and its derivatives has contributed to the rapid increase of *Plasmodium falciparum* strains (Gogoi *et al.* 2019). Artemisinin and its derivatives have saved millions of people suffering from malaria and have become the most effective drug against malaria (Yang *et al.* 2020). Currently, artemisinin-based combination therapies (ACTs) is the backbone of malaria control, especially in sub-Saharan Africa (Plucinski *et al.* 2015; Kumar *et al.* 2018). ACTs demonstrated high efficacy against all *Plasmodium falciparum* strains until recently when the number of treatment failures increased (Shibeshi *et al.* 2020; Chaniad *et al.* 2021). Therefore, there is a need to explore and find novel antimalarial agent to overcome the challenges associated with this drug resistance (Comer *et al.* 2014).

In this regard, medicinal plants are known to be a significant source of bioactive compounds that could serve as lead candidates for the development of new potent drugs (Dzouemo *et al.* 2022). For example, the genus *Diospyros* from the Ebenaceae family is one of the richest sources of chemical constituents. Various chemical compounds such as triterpenes, naphthoquinones, steroids and flavonoids have been isolated from different species of this genus (Rauf *et al.* 2015; Peyrat *et al.* 2016). The active compounds isolated from the species of this genus have demonstrated interesting biological activities, such as analgesic and anti-inflammatory activity ((Rauf *et al.* 2017), cytotoxic activity (Wisetsai *et al.* 2021), antimicrobial activity (Ayepola *et al.* 2018), alleviation of pain and fever (Adzu *et al.* 2002), antioxidant properties (Tameye *et al.* 2022), anthelmintic activity (Rathore *et al.* 2014), antidiabetic activity (Ruphin *et al.* 2014), antiproliferative activity (Feumo Feusso *et al.* 2019) and anti-malarial activity (Tangmouo *et al.* 2010; du Preez-Bruwer *et al.* 2022). In particular, *Diospyros lanceifolia* is one of the 700 species of the genus *Diospyros* of the Ebenaceae family (POWO 2025). This plant grows up to 27 m tall, with its stems bearing up to 10 flowers and twigs that are reddish brown when young and dark brown when mature (Malewska 2022). The plant was previously investigated for its phytochemical constituents, and so far, only four compounds have been reported from this plant, including plumbagin, lupeol and 7-methyl juglone, which were isolated from the 70% aqueous ethanolic extract of the leaves (Kichu 2015), as well as gallic acid isolated which was isolated from the aqueous methanolic extract of the leaves (Kalita *et al.* 2016; Malewska 2022). When assayed for biological activities, these compounds are reported to have strong antioxidant and antibacterial activities (Kalita *et al.* 2016; Malewska 2022).

Based on the previous report on this plant, this study aims to discover the chemical constituents present in the ethyl acetate (EtOAc) bark extract of *Diospyros lanceifolia* and evaluate their *in vitro* inhibitory activity against *Plasmodium falciparum* FCR3 strain followed by *in silico* molecular docking analysis on *Plasmodium falciparum* ATPase 6 (*Pf*ATP6) protein. The *in silico* study was performed to evaluate the binding interaction of the *Pf*ATP6 protein, which is a plausible target for antimalarial drugs (Diedrich *et al.* 2018; Moore *et al.* 2022). This is the first report on the chemical constituents of *Diospyros lanceifolia* assayed for their antiplasmodial activity and this study will provide further scientific information on the application of this species.

## MATERIALS AND METHOD

### General

All chemical reagents and solvents such as *n-*hexane, dichloromethane (DCM), EtOAc, methanol (MeOH), chloroform-D1, vanillin and sulphuric acids (AR grades) were obtained from QRëC (Asia) and Merck (Germany) used without further purification. Column chromatography (CC) was employed to separate fractions using silica gel 60 of 70–230 mesh and 230–400 mesh (Merck, Germany) as the stationary phase depending on the weight of the crude or fractions. To distinguish the presence of compounds in the extracts, thin-layer chromatography (TLC) was carried out on alumina plates pre-coated with silica gel 60 F_254_ plates (Merck, Germany). The TLC plates were examined using a UV radiation lamp (λ_max_ = 254 nm and 365 nm) and vanillin-sulphuric acid was used as detecting reagent to visualise the spots of compounds. All spectral data were analysed by spectroscopic instruments. Fourier-transform Infrared (FT-IR) was recorded using a Perkin Elmer ATR FT-IR spectrometer in the 600 cm^−1^ –4,000 cm^−1^ range. Also, Bruker Advance 500 (500 MHz for ^1^H-NMR, 125 MHz for ^13^C-NMR) spectrometer was used to record the 1D and 2D Fourier-transform nuclear magnetic resonance (FT-NMR) spectra in CDCl _3_ (^1^H: 7.26 ppm and ^13^C: 77.0 ppm) using tetramethyl silane (TMS) as internal standard. TopSpin 3.6.2 software package was used to analyse the data. Chemical shifts are recorded in parts per million (ppm) and coupling constants, *J* are presented in Hertz (Hz). The high-resolution electrospray ionisation mass spectrometry (HRESIMS) analysis of the compounds was recorded with a Water Xevo QTOF MS spectrometer and the data obtained are reported in *m/z*. Melting points were determined on open capillary tubes using the Stuart SMP-10 melting point apparatus. An Agilent BioTek Synergy H1 multimode reader was used to measure SYBR green fluorescence. Chloroquine and artemisinin were purchased from Sigma-Aldrich Chemical Company (Missouri, USA). SYBR Green I nucleic acid staining dye (10,000 × stock concentration) was purchased from Molecular Probes, Inc. (Oregon, USA) that stored frozen at 20°C and freshly thawed before use.

### Plants Material

The bark of *Diospyros lanceifolia* with code number KL5277B was collected in June 2007 at the Reserved Forest of Madek, Lenggor, Kluang, Johor, Malaysia. The plant specimen was identified by the botanist Teo L E, from Universiti Malaya. Afterwards, the voucher specimen was stored at the Herbarium of the Chemistry Department, Faculty of Science, Universiti Malaya, Kuala Lumpur, Malaysia.

### Extraction and Isolations of Chemical Constituents

The dry bark of *Diospyros lanceifolia* (1.0 kg) underwent extraction in 4.5 L EtOAc at room temperature for three consecutive days, to allow the solvent to extract the soluble molecule within their polarity. The process was repeated two times successively using fresh EtOAc solvent. The extracts were filtered on a No. 1 Whatman filter paper and the filtrates were evaporated using a rotary evaporator under a reduced pressure with a temperature below 40°C to obtain 17.9 g EtOAc crude extracts. The residue was then immersed in 4.5 L of MeOH by following a similar method above. The MeOH crude extracts were kept for future use. Next, 17.9 g of EtOAc crude extracts were subjected to CC over a silica gel 60 (70–230 mesh) eluted with *n*-hexane/EtOAc (100:0 → 20:80) gradient solvent system of increasing polarity to give five major fractions denoted as DL1 to DL5.

Based on the ^1^H-NMR spectra, DL1 to DL5 were subjected to further purification using CC by elution with *n*-hexane/EtOAc (100:0 → 20:80) step gradient solvent system. DL1 (1.56 g) was fractionated using CC in a gradient solvent system by elution with *n*-hexane/EtOAc (90:10) to afford compounds **1** (90 mg) and **2** (0.56 g). DL2 (3.1 g) was purified with CC and eluted with *n*-hexane/EtOAc (90:10), yielding compound **1** (2.8 g). Similarly, DL3 (2.2 g) was separated using the same method as above to give compounds **1** (0.45 g), **3** (20 mg), **4** (10 mg) and **5** (40 mg) with the *n*-hexane/EtOAc solvent system of (80:10), (85:15) and (80:20), respectively. In addition, the same purification protocol of DL4 (2.56 g) gave compounds **1** (10.02 g), **2** (32 mg) and **6** (80 mg) in the hexane/EtOAc solvent system of (80:10), (85:15) and (80:20), respectively. Furthermore, DL5 (1.26 g) was purified using CC and microcolumn that led to the isolation of compounds **7** (14 mg) and **8** (20 mg) in the (80:20) *n*-hexane/EtOAc solvent system. Notably, compound **1** was found to be a major constituent of this plant, followed by compound **2**. The structure of these compounds was determined using 1D and 2D-NMR, FTIR as well as HRESIMS and then compared with reported literature.

#### Lupeol (1)

White powder. Yield: 5.41 g (0.55%). M.p.: 219°C–221°C. FT-IR (ATR) v_max_ cm^−1^ : 3307 (O-H), 2936 (C_sp_^2^-H), 2861 (C_sp_^3^ -H), 1647 (C=C); HRESIMS (^+^ESI) [M+H]^+^ : 427.3927 (calcd for C_30_H_50_O, *m/z* 427.3759). ^1^H-NMR (500 MHz, CDCl_3_ ): 0.91 (m, H-1), 1.59, 1.65 (m, H-2), 3.18 (dd. J = 5.1,11.5 Hz, H-3), 0.67 (d, J = 9.5 Hz, H-5), 1.38, 1.51 (m, H-6), 1.38 (m, H-7), 1.26 (m, H-9), 1.41 (m, H-11), 1.65 (m, H-12), 1.64 (m, H-13), 1.65 (m, H-15), 1.36, 1.47 (m, H-16), 1.36 (m, H-18), 2.38 (m, H-19), 1.30, 1.91 (m, H-21), 1.37, 1.19 (m, H-22), 0.97 (s, H-23), 0.76 (s, H-24), 0.82 (s, H-H-25), 1.03 (s, H-26), 0.93 (s, H-27), 0.79 (s, H-28), 4.58, 4.67 (H-29), 1.67 (s, H-30). ^13^C-NMR (125 MHz, CDCl_3_ ): 38.9 (C-1), 27.5 (C-2), 79.1 (C-3), 39.0 (C-4), 55.5 (C-5), 18.5 (C-6), 34.4 (C-7), 41.1 (C-8), 50.6 (C-9), 37.3 (C-10), 21.1 (C-11), 25.3 ( C-12), 38.2 (C-13),43.0 (C-14), 27.6 (C-15), 35.8 (C-16), 43.1 (C-17), 48.5 (C-18), 48.1 (C-19), 151.0 (C-20), 30.0 (C-21), 40.1 (C-22), 28.1 (C-23), 15.5 (C-24), 16.2 (C-25), 16.1 (C-26), 14.7 (C-27), 18.1 (C-28), 109.5 (C-29), 19.4 (C-30).

#### Betulin (2)

White powder. Yield: 0.88 g (0.09%). M.p.: 242°C–246°C. FT-IR (ATR) v_max_ cm^−1^ : 3357 (O-H), 2939 (Csp^2^-H), 2867 (Csp^3^ -H), 1643 (C=C). HRESIMS (^+^ESI) [M+Na]^+^: 465.3709, (calcd for C_30_H_50_O_2_Na, *m/z* 465.3708). ^1^H-NMR (500 MHz, CDCl_3_ ): 0.88 (dd, J = 5.1, 12.5 Hz, H-1), 1.59, 1.64 (m, H-2), 3.20 (dd, J = 5.0, 11.5 Hz, H-3), 0.69 (d, J = 10.0 Hz, H-5), 1.39, 1.53 (m, H-6), 1.39 (m, H-7), 1.25 (m, H-9), 1.21, 1.42 (m, H-11), 1.05, 1.65 (m, H-12), 1.61 (m, H-13), 1.65, 1.70 (m, H-15), 1.28, 1.91 (m, H-16), 1.59 (m, H-18), 2.38 (m, H-19), 1.26, 1.96 (m, H-21), 1.03, 1.85 (m, H-22), 0.97 (s, H-23), 0.75 (s, H-24), 0.83 (s, H-25), 1.02 (s, H-26), 0.98 (s, H-27), 3.33 (d, J = 11.2 Hz, H-28), 4.58, 4.68 (H-29), 1.68 (s, H-30). ^13^ C-NMR (125 MHz, CDCl_3_ ): 38.9 (C-1), 27.5 (C-2), 79.2 (C-3), 39.0 (C-4), 55.5 (C-5), 18.5 (C-6), 34.4 (C-7), 41.1 (C-8), 50.5 (C-9), 37.3 (C-10), 20.9 (C-11), 25.2 (C-12), 37.3 (C-13), 42.9 (C-14), 27.2 (C-15), 29.2 (C-16), 47.9 (C-17), 48.9 (C-18), 47.9 (C-19), 150.3 (C-20), 29.8 (C-21), 34.2 (C-22), 28.2 (C-23), 15.5 (C-24), 16.2 (C-25), 16.1 (C-26), 14.9 (C-27), 60.8 (C-28),109.8 (C-29),19.2 (C-30).

#### *β*-Sitosterol (3)

White powder. Yield: 0.02 g (0.002%). FT-IR(ATR) v_max_ cm^−1^ : 3424 (O-H), 2922 (C_sp_^2^-H), 2847 (C_sp_^3^ -H), 1636 (C=C); HRESIMS (^+^ESI) [M+Na]^+^: 437.3769, (calcd for C_29_ H_50_ONa, *m/z* 437.38613). ^1^H-NMR (500 MHz, CDCl_3_ ): 1.47 (m, H-1), 2.1 (m, H-2), 3.54 (H-3), 2.33 (m, H-4), 5.37 (m, H-6), 2.03 (m, H-7), 1.66 (m, H-8), 1.21, 1.52 (m, H-9), 1.05, 1.49 (m, H-11), 1.52 (m, H-12), 1.50 (m, H-14), 1.59 (m, H-15), 1.85 (m, H-16), 1.47 (m, H-17), 1.26, 0.70 (s, H-18), 1.03 (s, H-19), 1.59 (m, H-20), 0.94 (s, H-21), 0.93 (m, H-22), 1.16 (m, H-23), 1.39 (m, H-24), 1.69 (m, H-25), 0.84 (d, J = 6.3 Hz, H-26), 0.85 (d, J = 6.3 Hz, H-27), 1.25 (m, H-28), 0.84 (s, H-29). ^13^ C-NMR (125 MHz, CDCl_3_ ): 37.2 (C-1), 31.7 (C-2), 71.9 (C-3), 42.4 (C-4), 140.8 (C-5), 121.8 (C-6), 32.0 (C-7), 32.0 (C-8), 50.2 (C-9), 36.6 (C-10), 21.1 (C-11), 39.8 (C-12), 42.3 (C-13), 56.8 (C-14), 24.4 (C-15), 28.3 (C-16), 56.1 (C-17), 11.9 (C-18), 19.5 (C-19), 36.2 (C-20), 18.8 (C-21), 34.0 (C-22), 26.1 (C-23), 45.9 (C-24), 29.2 (C-25), 19.9 (C-26), 19.1 (C-27), 23.1 (C-28),12.0 (C-29).

#### Oleic acid (4)

C_18_ H_34_O_2_; Yellowish white powder. Yield: 0.01 g (0.001%). FT-IR (ATR) v_max_ cm^−1^ : 3658 (O-H), 2914 (C_sp_^2^-H), 2839 (C_sp_^3^ -H), 1695 (C=C). ^1^H NMR (500 MHz, CDCl_3_ ): 2.36 (t, J = 7.5 Hz, H-2), 1.64 (m, H-3), 1.28–1.33 (m, H-4–H-7, H-12–H-17), 5.34 (m, H-9, H-10) 2.07 (m, H-8, H-11), 0.89 (t, J = 7.5 Hz, H-18). ^13^C NMR (125 MHz, CDCl_3_ ), 180.2 (C-1), 34.1 (C-2), 24.9 (C-3), 29.4 (C-4, 5, 13), 29.3 (C- 6), 29.7 (C-7, 12, 14), 29.2 (C-8), 130.0 (C-9), 129.8 (C-10), 27.2 (C-11), 29.6 (C-15), 31.9 (C-16), 22.7 (C-17), 14.1 (C-18).

#### *α*-Amyrin acetate (5)

C_32_ H_52_O_2_; A dark brown liquid. Yield: 0.014 g (0.0014%). FT-IR (ATR) v_max_ cm^−1^ : 2903 (C_sp_^2^-H), 2839 (C_sp_^3^ -H), 1738 (C=O), 1638 (C=C) cyclic methylene’s. ^1^H-NMR (500 MHz, CDCl_3_ ): 1.58 (m, H-1), 1.19 (m, H-2), 4.42 (dd, J = 14.4, 4.6 Hz, H-3), 0.76 (m, H-5), 1.45, 1.30 (m, H-6), 1.39, 1.26 (m, H-7), 1.46 (m, H-9), 1.86 (m, H-11), 5.20 (d, J = 12 Hz, H-12), 1.79 (m, H-15), 1.94 (m, H-16), 2.16 (m, H-18), 1.26 (m, H-19), 0.89 (m, H-20) 1.44 (m, H-21), 1.65 (m, H-22), 0.81 (s, H-23), 0.89 (s, H-24), 0.78 (s, H-25), 0.71 (s, H-26), 1.00 (s, H-27), 0.82 (s, H-28), 0.71 (s, H-29), 0.89 (s, H-30). ^13^ C-NMR (125 MHz, CDCl_3_ ): 38.3 (C-1), 29.1 (C-2), 81.0 (C-3), 39.5 (C-4), 55.3 (C-5), 18.2 (C-6), 32.9 (C-7), 42.0 (C-8), 47.5 (C-9), 37.0 (C-10), 23.3 (C-11), 125.6 ( C-12), 138.0 (C-13),48.0 (C-14), 28.0 (C-15), 24.1(C-16), 37.7 (C-17), 52.6 (C-18), 39.0 (C-19), 38.8 (C-20), 30.6 (C-21), 36.7 (C-22), 28.1 (C-23), 15.6 (C-24), 16.7 (C-25), 17.1 (C-26), 23.6 (C-27), 25.9 (C-28), 17.0 (C-29), 21.3 (C-30), 21.4 (C-31), 171.2 (C-32).

#### Glyceryl trilinoleate (6)

C_57_H_98_ O_6_ ; Yellow powder. Yield: 0.06 g (0.006%). FT-IR (ATR) v_max_ cm^−1^ : 2922 (C_sp_^2^-H), 2839 (C_sp_^3^ -H), 1736 (C=O) 1621 (C=C). ^1^H-NMR (500 MHz, CDCl_3_ ): 4.09 (dd, J = 12.1, 6.5 Hz, H-1), 4.24 (dd, J = 11.8, 4.1 Hz, H-3), 5.20 (m, H-2), 2.25 (t, J = 7.8 Hz, H-2), 1.53 (m, H-3), 1.18–1.22 (m, H-4–7), 1.95 (m, H-8), 5.29 (m, H-9), 5.30 (m, H-10), 2.72 (m, H-11), 5.29 (m, H-12), 5.30 (m, H-13), 2,05 (m, H-14), 1.18–1.22 (m, H-15–17), 0.88 (t, J = 7 Hz, H-18). ^13^C NMR (125 MHz, CDCl_3_ ), 62.1 (C-1, 3), 68.9 (C-2), 173.4 (C=1), 34.1 (C-2), 25.6 (C-3), 29.1–31.9 (C-4-C-7), 27.2 (C-8, C-14), 130.1 (C-9, C-13), 129.7 (C-10, C-12), 29.8 (C-15) 29.9 (C-16) 22.9 (C-17), 14.3 (C-18).

#### *β*- Amyrin (7)

C_30_H_50_O; White powder. Yield: 0.014 g (0.0014%). FT-IR (ATR) v_max_ cm^−1^ : 3282 (O-H), 2938 (C_sp_^2^-H), 2839 (C_sp_^3^ -H), 1719 (C=C) cyclic methylene’s, 1460. ^1^H-NMR (500 MHz, CDCl_3_ ): 1.63, 0.99 (m, H-1), 1.60, 0.77 (m, H-2), 3.23 (dd, J = 11.1, 4.6 Hz, H-3), 0.75 (m, H-5), 1.42, 1.52 (m, H-6), 1.33, 1.50 (m, H-7), 1.53 (m, H-9), 1.86 (m, H-11), 5.18 (t, J = 7.0 Hz, H-12), 0.96, 1.75 (m, H-15), 1.98 (m, H-16), 0.94 (m, H-18), 1.01, 1.64 (m, H-19), 1.09, 1.32 (m, H-21), 1.21, 1.40 (m, H-22), 0.99 (s, H-23), 0.78 (s, H-24), 0.90 (s, H-25), 0.96 (s, H-26), 1.13 (s, H-27), 0.82 (s, H-28), 0.86 (s, H-29), 0.86 (s, H-30). ^13^ C-NMR (125 MHz, CDCl _3_ ): 38.6 (C-1), 27.3 (C-2), 71.1 (C-3), 39.8 (C-4), 55.2 (C-5), 18.4 (C-6), 32.7 (C-7), 38.8 (C-8), 47.7 (C-9), 37.0 (C-10), 23.6 (C-11), 121.8 ( C-12), 145.3 (C-13),41.8 (C-14), 26.2 (C-15), 27.0 (C-16), 32.6 (C-17), 47.3 (C-18), 46.9 (C-19), 31.2 (C-20), 34.8 (C-21), 37.2 (C-22), 28.2 (C-23), 15.7 (C-24), 15.6 (C-25), 16.9 (C-26), 26.1 (C-27), 28.5 (C-28), 33.4 (C-29), 23.8 (C-30).

#### Shinanolone (8)

Brown powder. Yield: 0.02 g (0.002%). FT-IR (ATR) v_max_ cm^−1^ : 3307 (O-H), 2930 (C_sp_^2^-H), 2831 (C_sp_^3^ -H), 1719 (C=O) 1636 (C=C), 1452 cyclic methylene. HRESIMS (^−^ESI) [M-H]^−^: 190.9283, (calcd for C_11_ H_11_ O_3,_ *m/z* 190.0709). ^1^H NMR (500 MHz, CDCl_3_ ): 2.55, 2.89 (m, H-2), 2.18, 2.91 (m, H-3), 4.81 (dd, J = 8.5, 3.5 Hz, H-4), 6.79 (s, H-5) 6.69 (s, H-7), 2.30 (s, CH_3_ ), 12.35 (OH). ^13^C NMR (125 MHz, CDCl_3_ ): 203.5 (C-1), 34.5 (C-2), 31.5 (C-3), 67.8 (C-4), 118.6 (C- 5), 145.6 (C-6), 117.7 (C-7), 162.8 (C-8), 113.2 (C-8a), 148.8 (C-4a), 22.3 (CH_3_ ).

### Antiplasmodial Activity

#### Plasmodium Culture

The strains of *Plasmodium falciparum* FCR3 were cultured using the modified Trager and Jensen (1976) method. The *Plasmodium* parasite was cultured in human O red blood cells in RPMI 1640 medium supplemented with 10% human O serum. The RPMI medium was made by adding 10.0 g of RPMI 1640 powder, 25 mM HEPES buffer, L-glutamine, 2 g of sodium bicarbonate (NaHCO_3_ ) and distilled water up to 1 L. The pH medium was adjusted to ±7.2 and was sterilised using a 0.22 μM filter stored at 2°C–8°C. Then *Plasmodium* culture was incubated at a temperature of 37°C and was observed for every 24 h until the parasite was ready for the assay with a minimum parasitemia of 2%. Subsequently, the stock culture was synchronised with 5% D-sorbitol to produce the ring stage.

### Antiplasmodial Activity Using Microfluorescence SYBR^®^ Green

The antiplasmodial assay was conducted based on the method of Smilkstein *et al.* (2004) with slight modification. The parasite solution for testing contains RPMI medium complemented with 10% human serum (HS), 2% parasitemia and 2% hematocrit. Then 100 μL/well of *Plasmodium* suspension was added into the 96-well microplate. Subsequently, all testing compounds in various concentrations (100 μg/mL, 50 μg/mL, 25 μg/mL, 12.5 μg/mL and 6.2 μg/mL) with a volume of 100 μL/well were incorporated into each well of the parasite solution. Also, as much as 100 μL of standard drug, media control and 1% DMSO solvent control were added 100 μL to each well of the parasite solution. Then, 200 μL of 2% RBC control was added to the designated wells without administering parasite solution. Each series of concentrations was made in three replicates and the test was repeated three times. Subsequently, it was incubated at 5% CO_2_ atmosphere at 37°C for 48 h. After the incubation process, the parasite solution was transferred to a microtube to be centrifuged for 5 min at 1,600 rpm, then washed once using 1 mL of 1x PBS, then the parasite solution was placed in a −20°C freezer overnight. The parasite solution was then removed from the freezer and left for approximately 3 h at room temperature. This process is a freeze-thaw process that aims to lyse cells. Following this, SYBR green 2x 100 μL/well is added to a dark well plate for staining in a dark room. Carefully avoiding contact with the SYBR green, the homogenised parasite solution is transferred to the well plate. Afterward, the plate is incubated at room temperature for 1.5 h, covered with aluminium foil and stored in a dark area. The SYBR Green fluorescence reading, using an excitation wavelength of 485 nm and emission wavelength of 528 nm, was carried out with a multimode reader (Agilent BioTek Synergy H1) to determine the percentage of inhibition based on the relative fluorescence unit (RFU) value obtained from the readings.

In the fluorescence assay method with SYBR green, the RFU value is calculated which describes the interaction of parasite DNA with SYBR green. The RFU obtained from the reading results uses the following equation:


(1) $$\text{Proteolytic index}=100-\frac{\text{Sample RFU}-\text{RBC control RFU}}{\text{RPMI control RFU}-\text{RBC control RFU}}\times 100\%$$

### Statistical Analysis

All experiments were conducted in triplicate and the data obtained from the analyses were expressed as mean ± standard deviation (SD) with (*n* = 3). The IC_50_ value was calculated by probit analysis using the Statistical Package for Social Sciences (SPSS) software package (IBM Corp., Chicago, USA). The lower the half-maximal inhibitory concentration (IC_50_) value obtained, the better the antiplasmodial activity.

### Molecular Docking

The structures of the isolated compounds identified as ligands were drawn using ChemDraw Professional 22.00 software (Perkin Elmer Informatics, Massachusetts, USA). The ligands were then transformed into three-dimensional (3D) structures using the Chem3D tools in the ChemDraw software. The energy was minimised and the structure was optimised using the MM2 force field. The structures were then saved in the protein databank (.pdb) format. The homology model of the *Pf*ATP6 protein was obtained from the Swiss Institute of Bioinformatics (https://www.modelarchive.org/doi/10.5452/ma-cies5) (Salas-Burgos *et al.* 2004). The preparation of the protein was performed using BIOVIA Discovery Studio Visualiser 2021 (Dassault Systems, California, USA) including removing the heteroatoms comprising water molecules and other associated inhibitors around the crystal structure of the protein and later saved in a .pdb file. The DockPrep tools in the UCSF Chimera software (Regents of University of California, USA) were used to generate the .pdbqt file for docking preparation. During this process, various adjustments were made to the ligands and protein, such as adding polar hydrogen atoms, merging non-polar hydrogen atoms, setting solvation parameters and calculating Gasteiger charges. In addition, these adjustments aim to improve the affinity of the protein binding site (Dzouemo *et al.* 2022). The docking parameters were set with a grid box size of 28 × 28 × 28 and a default grid spacing of 0.375 Å with coordinates of 59.32, 15.12 and 18.71 for X, Y and Z, respectively. The docking experiment was performed with AutoDock Vina 1.5.7 (Trott & Olson 2010; Eberhardt *et al.* 2021) in three independent molecular docking runs. This docking experiment was performed with all isolated compounds as well as with standard drugs (artemisinin and chloroquine), which served as a guide to validate the docking method by benchmarking. After completion of the docking simulation, 10 ligand-protein models were generated and evaluated based on binding affinity. Finally, the ligand-receptor model that exhibited the most favourable binding energy and best interactions was selected for further visualisation and analysis of their active interactions in 2D and 3D conformations, also using BIOVIA Discovery Studio Visualiser 2021 (Dassault Systems, California, USA).

## RESULTS AND DISCUSSION

**Figure 1:**
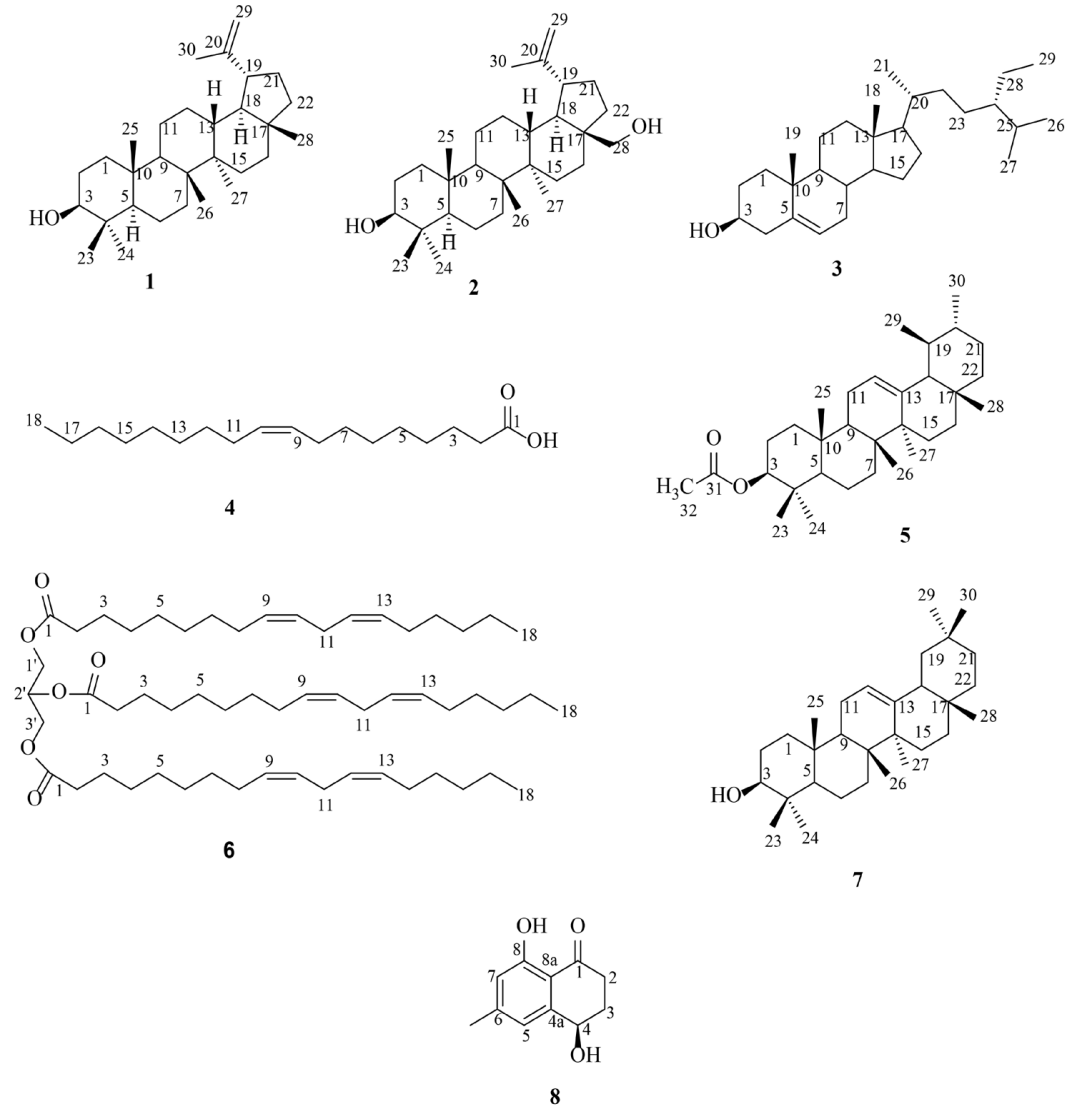
Chemical structure of the isolated compounds from the bark of *Diospyros lanceifolia* .

The chemical investigations of the EtOAc bark extract of *Diospyros lanceifolia* have led to the isolation of eight compounds. The structures of these compounds (Fig. 1) were established using various spectroscopic techniques such as 1D and 2D-NMR, FT-IR and HRESIMS, which then further compared with reported literature to support elucidation. These eight isolated compounds are identified as lupeol (**1**) (Bafarawa *et al.* 2024), betulin (**2**) (Ghani *et al.* 2022), *β-*sitosterol (**3**) (Nweze *et al.* 2019), oleic acid (**4**) (Ahmed *et al.* 2021), *α*-amyrin acetate (**5**) (Karan *et al.* 2013), glyceryl trilinoleate (**6**) (Ahmed *et al.* 2021), *β-*amyrin (**7**) (Thing *et al.* 2014) and shinanolone (**8**) (Aung *et al.* 2002).

### Antiplasmodial Activity Results

In view of the emergence of drug resistance to ACTs and the search for new compounds that could substitute the currently used antimalarial drugs, the compounds isolated from the EtOAc bark extract of *Diospyros lanceifolia* were investigated for *in vitro* antimalarial studies. In particular, these eight compounds isolated from this plant were tested for their *in vitro* antiplasmodial activity against the *Plasmodium falciparum* FCR3 strain, using artemisinin and chloroquine as reference drugs. To confirm the activity of a tested compound, Sikam *et al.* (2022) reported that an isolated compound can be classified as active if its IC_50_ value from in vitro analysis is below 10 μM. Therefore, the IC_50_ values for each of the tested compounds, including the controls, are listed in Table 1.

Based on these criteria, five out of the eight compounds isolated are considered active with values below 10 μM. In order of potency, these compounds include *α-*amyrin acetate (**5**), *β*-sitosterol (**3**), glyceryl trilinoleate (**6**), lupeol (**1**) and oleic acid (**4**), respectively. Compounds **1** and **5** are pentacyclic triterpenoids and have demonstrated strong activity against *Plasmodium falciparum* FCR3. Previous research has shown that pentacyclic triterpenoids, including betulinic acid and ursolic acid along with their derivatives, have moderate to strong antimalarial activity compared to standard chloroquine (Abd Ghani *et al.* 2024). Nevertheless, the exact mechanisms by which triterpenoids exert their antimalarial effect are not yet fully understood (da Silva *et al.* 2013). In addition, ursolic acid derivatives that have an acetyl group at the C-3 position show significant antimalarial activity (Gnoatto *et al.* 2008). Considering that the active pentacyclic triterpenoids in this study share an almost identical structural scaffold with ursolic acid and betulinic acid, it was expected in this study that these triterpenoids would exhibit comparable antimalarial activity due to a similar mechanism.

Compound **1** is classified as a pentacyclic triterpenoid that belongs to the lupane series. This compound has demonstrated significant *in vitro* antiplasmodial activity with an IC_50_ value of 4.4 ± 7.4 μM. The *in vitro* activity of compound **1** was lower than that of artemisinin but higher than that of chloroquine. The findings of the present study are in agreement with previous literature, which indicated that compound **1** has significant inhibitory activity against *Plasmodium falciparum* 3D7 strain (Singh *et al.* 2020). Furthermore, Masia *et al.* (2024) suggested that the antiplasmodial effect of compound **1** is characterised by its incorporation into the cell membrane of host erythrocytes, resulting in changes that inhibit parasite invasion rather than directly inducing toxicity in the malaria parasite. Next, compound **5** is classified as an ursane-type pentacyclic triterpenoid and has been associated with potential chemo preventive properties as well as efficacy in combating various health problems (Machado *et al.* 2018). In this study, compound **5** exhibited the strongest activity with an IC_50_ value of 0.3 ± 0.3 μM compared to the reference drugs artemisinin and chloroquine (IC_50_: 0.7 ± 0.3 μM and 10.3 ± 2.9 μM, respectively). The strong inhibitory effect of this compound could be related to its chemical properties and structure, in particular the presence of an ester group. This result is consistent with the aforementioned literature, which indicates that the acetyl group in the C-3 position of triterpenoids influences the activity of a compound (Gnoatto *et al.* 2008). However, the similar pentacyclic triterpenoid compounds of **7** and **2** showed moderate to weak antiplasmodial activity, respectively. The moderate activity of compound **7** is reflected in its IC_50_ value of 15.6 ± 19.9 μM, while compound **2** has an IC_50_ value above 50 μM (88.5 ± 14.5 μM), indicating weak *in vitro* activity against *Plasmodium falciparum* FCR3. In fact, a previous study by Karagöz *et al.* (2019) showed that compound **2** is inactive against *Plasmodium falciparum* 3D7 in comparison with the control drug chloroquine, which is consistent with the present study.

Meanwhile, compound **3** is a phytosterol found in many plants and has been studied for its diverse pharmacological activities, including *in vitro* activity against malaria. This compound has previously been described with considerable efficacy and has been categorised as a potent inhibitor against strains of *Plasmodium falciparum* . This activity was hypothesised to be due to its chemical properties and structural similarity to chloroquine (Yusuf *et al.* 2023). Indeed, compounds **3** and **5** in the present study had similar antiplasmodial activity with the IC_50_ value of 0.3 ± 0.3 μM, indicating higher activity compared to artemisinin and chloroquine. Furthermore, compounds **4** and **6** are fatty acids, which have been reported to reduce the activity of the malaria parasite due to the presence of a fatty acid side chain (Fotie *et al.* 2006). Besides, the fatty acid compounds have non-toxic properties and act as potent inhibitors of the fatty acid biosynthetic machinery of the *Plasmodium falciparum* parasite. Moreover, the efficacy of unsaturated fatty acids improves with increasing the degree of unsaturation (Melariri *et al.* 2012). In the current research, compounds **4** and **6** demonstrated good to strong antiplasmodial activity, with the IC_50_ of 8.4 ± 4.9 μM and 1.9 ± 2.2 μM, respectively. These compounds showed significant inhibitory activity, although their *in vitro* activity was lower than that of artemisinin but higher than that of the chloroquine control drugs. On the other hand, compound **6** showed higher activity than compound **4** due to the structural nature of compound **6**, which is a polyunsaturated fatty acid consisting of three linoleic acid moieties. This structural behaviour could be the reason for the strong inhibitory effect of this compound as described in the previously mentioned literature. Finally, compound **8** is a phenolic compound with the lowest antiplasmodial activity (IC_50_: 63.3 ± 10.8 μM). This compound is considered inactive as it had an IC_50_ value above 50 μM as well as six times higher than the standard criteria (<10 μM).

In conclusion, the five potent compounds isolated from *Diospyros lanceifolia* could be a potential lead candidate for the development of new antimalarial drugs. However, to establish them as viable antimalarials, a comprehensive study including molecular dynamics and *in vivo* testing is required to further validate the potential of these compounds as antimalarial agent.

### Molecular Docking Studies

In this study, the *in silico* molecular docking simulation of five potent compounds on the previous *in vitro* antiplasmodial activity was performed to investigate their binding affinity and to explore how these compounds interact with the homology model of *Pf*ATP6 protein. This protein, also known as *Pf*SERCA, consists of 1228 amino acids that share 51% (Range 2) similarity with the European rabbit SERCA protein (PDB ID: 1SU4) (Dohutia *et al.* 2017). The *Pf*ATP6 protein has no ligand, as its crystal structure has not yet been fully resolved in a database. Since the structure of the *Pf*ATP6 protein has not yet been solved, a template of the European rabbit SERCA protein mentioned above was used as a ligand reference to obtain the coordinates and grid box for the docking process of *Pf*ATP6 (Diedrich *et al.* 2018). Noteworthy, the major specific binding pocket or active site of *Pf*ATP6 protein includes LEU263, PHE264, GLN267, ILE977, ILE981, ALA985, ASN1039, LEU1040 and ILE1041, which are the important target for understanding the antiplasmodial activity of the tested compounds (Tsamesidis *et al.* 2022).

Therefore, the molecular docking study of the tested compounds (also known as ligands) consisting of five potent compounds as well as the standard drugs artemisinin and chloroquine, against the *Pf*ATP6 protein was computationally evaluated. The binding energy of each ligand-protein configuration was analysed and the docking poses (also known as model) with the lowest energy were selected as the best among the poses. The lower the binding energy, the better the binding affinity (Morris *et al.* 2009). The binding energy of the tested compounds with the *Pf*ATP6 protein is shown in Table 2.

**Figure 2:**
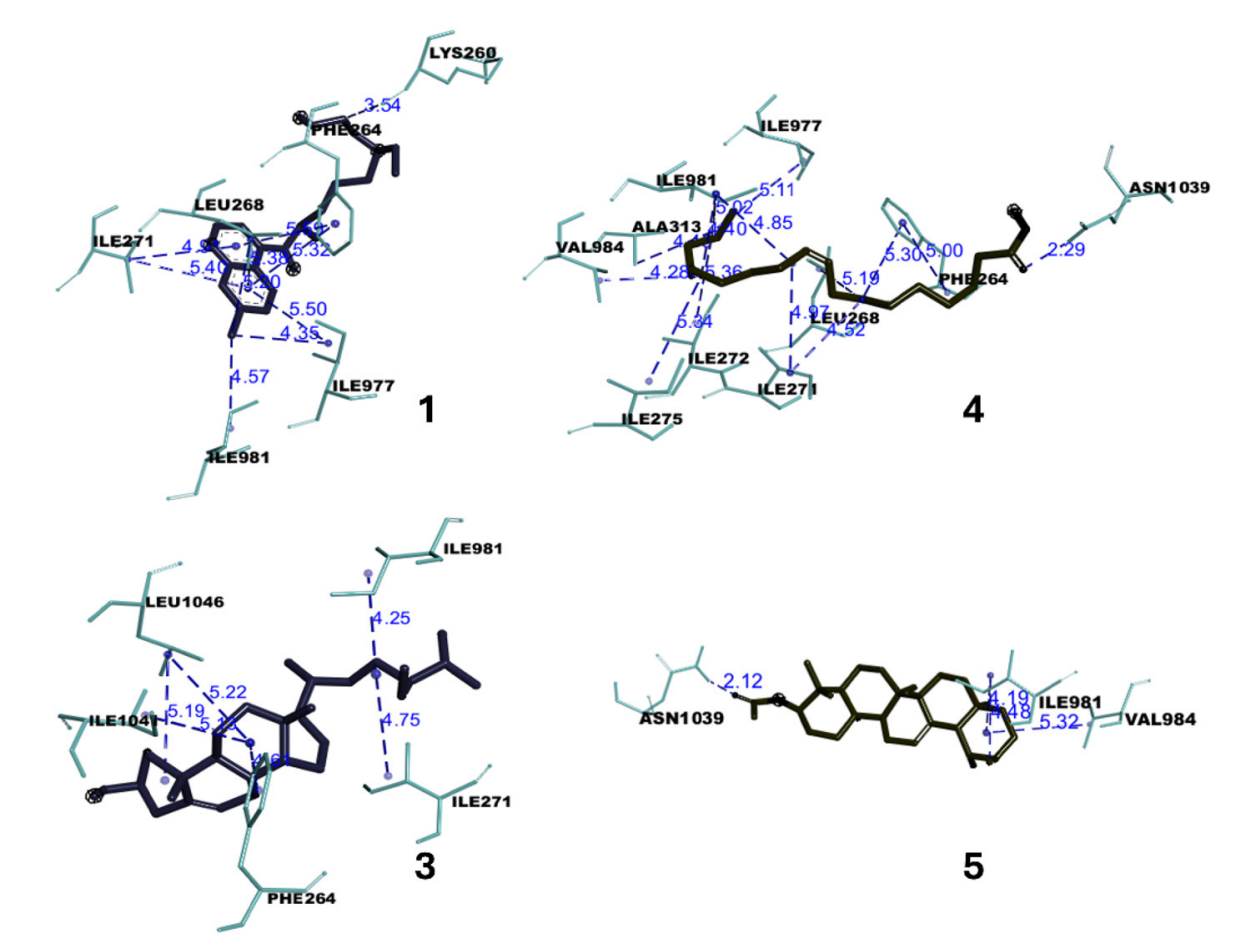
Illustration of 3D binding interactions of compounds **1**,**3**,**4** and **5** with *Pf*ATP6 protein.

**Figure 3:**
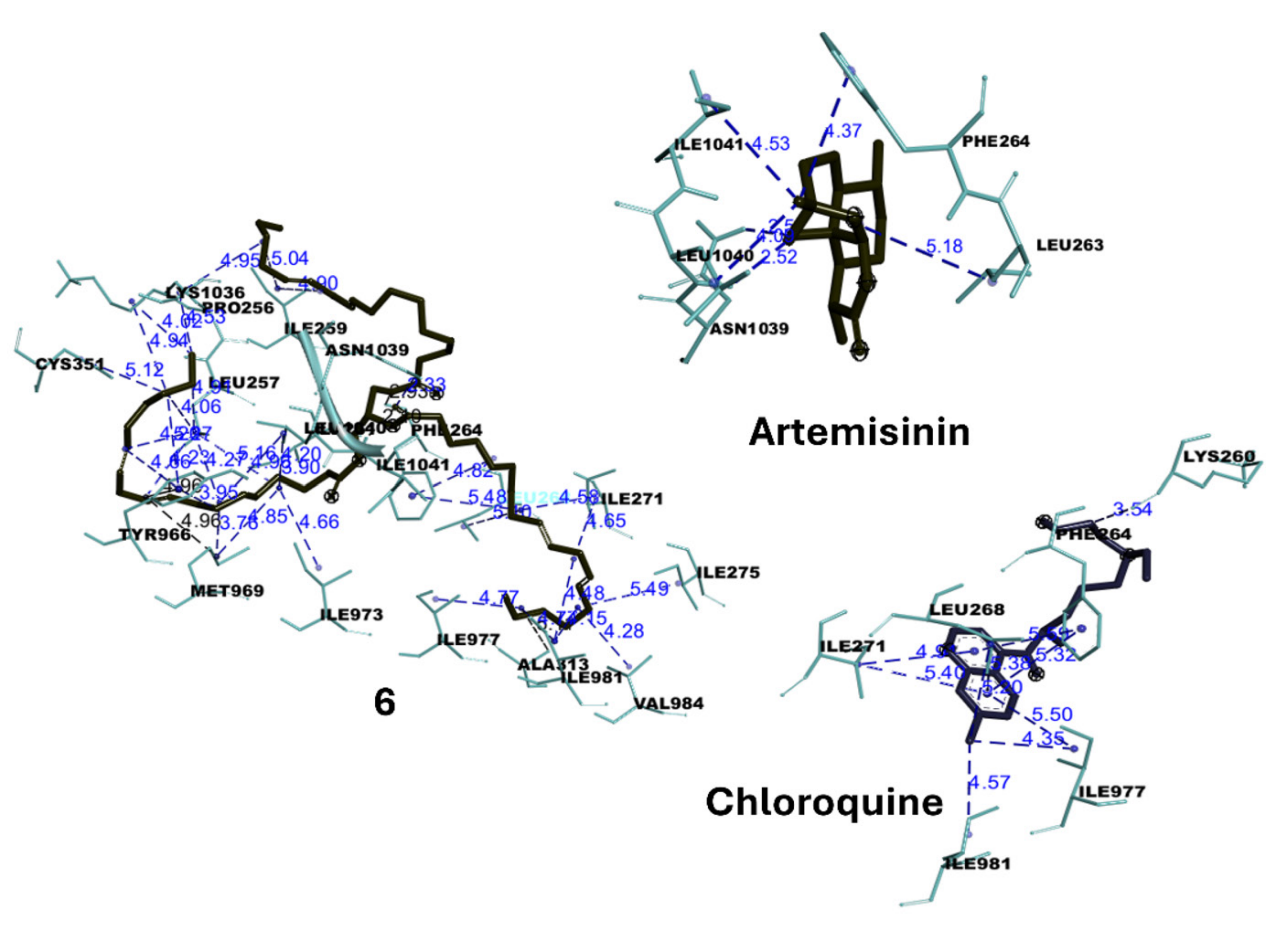
Illustration of 3D binding interactions of compounds **6**, artemisinin and chloroquine with *Pf*ATP6 protein.

According to the result in Table 2, all tested compounds showed good binding affinity with *Pf*ATP6 protein as the binding energies are below the threshold value of −5.0 kcal mol^−1^ , indicating a favourable interaction between them (Takahashi *et al.* 2010). Furthermore, lower binding energy is crucial in molecular docking studies as it directly correlates with stronger ligand-receptor binding and also indicates a thermodynamically favourable interaction, meaning that the ligand binds more tightly and stably to the target protein or receptor (Morris *et al.* 2009). All tested compounds establish binding interactions with residues LEU263, PHE264, GLN267, ILE977, ILE981, LEU1040 and ILE1041, which are the major specific binding pocket or active site of *Pf*ATP6 protein. The results of the *in silico* study of the five potent compounds significantly support the *in vitro* antiplasmodial study. The ligand-protein docking interactions are tabulated in Table 3, while the docked conformations of *Pf*ATP6 protein with ligands **1**, **3**, **4**, **5**, **6**, artemisinin and chloroquine positive controls are shown accordingly in Figs. 2 and 3 and later discussed.

Compound **1** is a pentacyclic triterpenoid that possesses a hydroxyl group at the C-3 position and has a binding energy of −8.1 kcal mol^−1^ , compared to the control drugs artemisinin and chloroquine with a binding energy of −7.4 ± 0.1 and −6.4 ± 0.3 kcal mol^−1^ , respectively. Compound **1** forms a hydrogen bond with the *Pf*ATP6 protein, which occurs between the hydroxyl group of **1** and the ASN1039 residues at a distance of 2.26 Å. In fact, hydrogen bonds play a crucial role in molecular docking by increasing the stability of the ligand-receptor complex and influencing the selectivity and specificity of ligand binding. The formation of hydrogen bonds with key receptor amino acids can significantly affect binding affinity and specificity, which contribute to a lower binding energy of the docked ligand-receptor (Trott & Olson 2010). Additionally, compound **1** participated in several hydrophobic alkyl interactions involving the cyclopentyl ring and the methyl groups of the isopropenyl moiety with the *Pf*ATP6 residues of ILE271, LEU1046 and ILE1050. Next, compound **3** is the naturally occurring steroid with the lowest binding energy of all compounds tested with a value of −8.3 kcal mol^−1^ . Compound **3** exhibited hydrophobic pi-alkyl interactions between the methylene groups of the cyclic ring (ring C) and the protein residues PHE264, ILE1041 and LEU1046. In addition, alkyl interactions were also detected between the non-ring methylene groups and the residues of ILE271 and ILE981. However, this compound showed no hydrogen bond interaction with the *Pf*ATP6 protein, but demonstrated strong activity in the *in vitro* antiplasmodial study (IC_50_: 0.3 ± 0.3 μM). This strong activity of this compound could be influenced by the lowest binding energy in combination with the detected alkyl and pi-alkyl interactions with the amino acid of the *Pf*ATP6 protein. This type of hydrophobic interaction results in a charge transfer that supports the intercalation of compound **3** into the active site of the *Pf*ATP6 protein (Abd Ghani *et al.* 2024).

Moreover, compound **5** is a ursane-type triterpenoid exhibiting strong antiplasmodial activity (IC_50_: 0.3 ± 0.3 μM) similar to that of compound **3**. Computational docking showed that compound 5 has a good binding energy of −7.9 ± 0.0 kcal mol^−1^ , comparable to the control drugs artemisinin and chloroquine. Compound **5** displayed conventional hydrogen bond with an amino acid ASN1039 via the oxygen of the ester group with a distance of 2.12 Å. In addition, a series of hydrophobic alkyl interactions were also formed between the methyl of the isopropyl group (at C-30) and the protein residues of ILE981 and between the methylene of ring E and the amino acid residues of ILE981 and ILE984. This good ligand-protein binding energy of this compound can be attributed to the aforementioned hydrophobic interactions with the amino acid residues of the *Pf*ATP6 protein. Besides, compound **4** is a fatty acid with a binding energy of −6.7 kcal mol^−1^ . Compound **4** presented a hydrogen bond with the amino acid ASN1039 at a distance of 2.29 Å through the hydroxyl moiety of the carboxylic acid group in the fatty acid chain. Also, several hydrophobic intercalations, including six alkyl and three pi-alkyl interactions were displayed between the methylene groups of the fatty acid chain and the residues of *Pf*ATP6, which also contributes to the good *in vitro* antiplasmodial activity (IC_50_ of 8.4 ± 4.9 μM) of this compound. For compound **6**, which is also a fatty acid, this compound exhibited the highest binding energy compared to the other compounds tested, including the control drugs, with a value of −5.8 ± 0.0 kcal mol^−1^ . This compound demonstrated three hydrogen bonds between the oxygen atom of the ester (at position C-1) and the residues PHE264, ASN1039 and ILE 1041. In addition to these hydrogen bonds, compound **6** also showed several hydrophobic interactions, including ten alkyl, six pi-alkyl and one pi-sigma interactions, mainly via the methylene group in its main straight fatty acid chain, which supported the binding of this compound to the surface of the *Pf*ATP6 protein, resulting in the strong *in vitro* activity of compound **6**.

Moving on to the control drug, artemisinin is a sesquiterpene lactone isolated from *Artemisia annua*. This compound and its derivatives are a fast-acting drug that targets the blood stage of *Plasmodium falciparum* and is also considered the most useful drug for the control of malaria (Tsamesidis *et al.* 2022). Molecular docking analysis revealed a binding energy of −7.4 ± 0.1 kcal mol^−1^ , which is very similar to that of compound **5**. Artemisinin formed two hydrogen bonds with the amino acids ASN1039 and LEU1040 across the oxygen atom attached to ring A with a distance of 2.52 and 2.50 Å, respectively. Similarly, it also shows two alkyl and two pi-alkyl interactions via the methyl groups of rings A and C with residues LEU263, PHE264, LEU1040 and ILE1041. Another control drug, chloroquine, a first-line antimalarial, had a binding energy of −6.4 kcal mol, a similar value to compound **4** (−6.7 ± 0.3 kcal mol^−1^ ). Chloroquine manifested a carbon-hydrogen bonding through an amine group attached to the compound. Besides, three alkyl, three pi-alkyl and two pi-pi T-shaped interactions were found between the chlorine moiety and quinoline rings of the compound with the *Pf*ATP6 residues. On the whole, it was found that all tested compounds in this *in silico* molecular docking analysis showed stability and binding affinity through hydrogen bonds (conventional hydrogen bond and carbon hydrogen bond) and hydrophobic interactions (alkyl, pi-alkyl, pi-sigma and pi-pi T-shaped) with the active site of *Pf*ATP6, which can be used as a plausible criterion to explain the findings of *in vitro* antiplasmodial activity in this present study.

## CONCLUSION

In conclusion, the phytochemical investigation of the bark of *Diospyros lanceifolia* led to the identification of eight compounds. Their structure was elucidated using various spectroscopic techniques and the data were compared with the reported literature for confirmation. The compounds were identified as pentacyclic triterpenoids, steroids, fatty acids and phenolic compounds. The results of *in vitro* antiplasmodial activity showed that five compounds were active in order of potency (**5**, **3**, **6**, **1** and **4**) with IC_50_ values of less than 10 μM. Furthermore, *in silico* assessment revealed that all tested compounds supported the *in vitro* result and showed good binding affinity to the binding site of *Pf*ATP6 protein. Compounds **3**, **1** and **5** showed a higher affinity and lower binding energy (−8.3 kcal mol^−1^ , −8.1 kcal mol^−1^ and −7.9 kcal mol^−1^ , respectively) compared to the control drugs of artemisinin and chloroquine (–7.3 kcal mol^−1^ and −6.5 kcal mol^−1^ , respectively). All tested compounds, with the exception of compound **3**, form hydrogen bonds with the amino acid ASN1039 of the *Pf*ATP6. These hydrogen bonds in combination with a series of hydrophobic interactions indicate that all tested compounds were able to stably bind to the active site of *Pf*ATP6. This *in silico* study provides deeper insights into how these compounds interact with the *Pf*ATP6 residues. Therefore, these compounds could be the potential lead candidates for the development of new antimalarial drug. To confirm the potential of these compounds, *in vivo* tests should be performed to further validate the possibility of these compounds as antimalarial agents.

## Supplementary Information



## Figures and Tables

**Table 1:** Activity of the compounds against *Plasmodium falciparum* FCR3.

Compound	IC_50_ (μM)*
1	4.4 ± 7.4
2	88.5 ± 14.5
3	0.3 ± 0.3
4	8.4 ± 4.9
5	0.3 ± 0.3
6	1.9 ± 2.2
7	15.6 ± 19.9
8	63.3 ± 10.8
Artemisinin	0.7 ± 0.3
Chloroquine	10.3 ± 2.9

Note.

* Mean ± standard deviation from at least three independent assays.

**Table 2:** *In silico* binding energy of tested compounds with homology model of *Pf*ATP6 protein.

Ligand	Binding energy (kcal mol^−1^)*
1	−8.1 ± 0.0
3	−8.3 ± 0.0
4	−6.7 ± 0.3
5	−7.9 ± 0.0
6	−5.8 ± 0.0
Artemisinin	−7.4 ± 0.1
Chloroquine	−6.4 ± 0.3

Note.

* Mean ± standard deviation from at least three independent molecular docking.

**Table 3:** Binding interactions between the potent compounds and control drugs with *Pf*ATP6 protein.

Ligand	Interacting unit of ligand	Protein interaction	Type of interaction	Distance (Å)
**1**	-OH	ASN A:1039	Conventional hydrogen bond	2.26
	-CH_3_ (Isopropenyl)	ILE A: 1050	Alkyl	5.09
	-CH_3_ (Isopropenyl)	LEU A :1046	Alkyl	4.23
	-CH_2_ Ring E	ILE A: 271	Alkyl	4.58

**3**	-CH_2_ Ring C	ILE A: 1041	Pi-alkyl	5.13
	-CH_2_ Ring C	LEU A:1046	Pi-alkyl	5.22
	-CH_2_ Ring C	PHE A: 264	Pi-alkyl	4.25
	-CH_2_	ILE A: 271	Alkyl	4.75
	-CH_2_	ILE A: 981	Alkyl	5.09

**4**	**-**OH	ASN A:1039	Conventional hydrogen bond	2.29
	-CH_2_	PHE A:264	Alkyl	5.00
	-CH_2_	LEU A:268	Alkyl	4.85
	-CH_2_	ILE A:271	Alkyl	5.30
	-CH_2_	ILE A: 275	Alkyl	5.34
	-CH_2_	ILE A:981	Alkyl	5.02
	-CH_2_	VAL A: 984	Alkyl	4.29
	-CH_2_	ILE A: 977	Pi-alkyl	5.11
	-CH_2_	ILE A: 272	Pi-alkyl	4.40
	-CH_2_	ALA A: 313	Pi-alkyl	4.45

**5**	O of ester	ASN A:1039	Conventional hydrogen bond	2.12
	-CH_2_ Ring E	VAL A: 984	Alkyl	5.91
	-CH_2_ Ring E	ILE A: 981	Alkyl	4.48
	-CH_3_	ILE A: 981	Alkyl	4.19

**6**	-O of ester	ILE A: 1041	Conventional hydrogen bond	2.90
	-O of ester	ASN A:1039	Conventional hydrogen bond	2.23
	-O of ester	PHE A: 264	Conventional hydrogen bond	2.93
	-CH_2_	PRO A:256	Alkyl	5.04
	-CH_2_	1LE A: 973	Alkyl	4.46
	-CH_2_	ILE A: 261	Alkyl	4.85
	-CH_2_	LEU A:1040	Alkyl	4.96
	-CH_2_	ILE A: 259	Pi-alkyl	
	-CH_2_	LEU A: 257	Pi-alkyl	5.49
	-CH_2_	TYR A: 966	Pi-sigma	4.66
	-CH_2_	MET A: 969	Alkyl	4.85
	-CH_2_	CYS A: 351	Alkyl	5.12
	CH_3_	LYS A:1036	Pi-alkyl	4.92
	-CH_2_	LEU A: 268	Pi-alkyl	5.10
	-CH_2_	ILE A: 271	Pi-alkyl	4.58
	-CH_2_	VAL A: 984	Alkyl	4.23
	-CH_2_	ILE A: 977	Pi-alkyl	4.77
	-CH_2_	ILE A:981	Alkyl	4.15
	-CH_2_	ALA A: 313	Alkyl	5.11
	-CH_2_	ILE A: 1041	Alkyl	3.95

Artemisinin	-O Ring A	LEU A:1040	Conventional hydrogen bond	2.50
	-O Ring A	ASN A: 1039	Conventional hydrogen bond	2.52
	-CH_3_ Ring A	LEU A: 1040	Pi-alkyl	4.09
	-CH_3_ Ring A	PHE A: 264	Alkyl	4.37
	-CH_3_ Ring A	ILE A:1041	Pi-alkyl	4.53
	-CH_2_ Ring C	LEU A: 263	Alkyl	4.09

Chloroquine	-Cl	ILE A:981	Alkyl	4.57
	-Cl	ILE A: 977	Pi-alkyl	4.50
	Quinoline Ring A	PHE A: 264	Pi-pi T-shaped	5.59
	Quinoline Ring B	LEU A:268	Alkyl	5.40
	-NH	LYS A: 260	Carbon hydrogen bonding	5.32
	Quinoline Ring B	ILE A: 271	Pi-alkyl	4.94
	-Cl	LEU A:268	Alkyl	5.00
	Quinoline Ring A	ILE A: 271	Pi-alkyl	4.94
	Quinoline Ring A	PHE A: 264	Pi-pi T-shaped	5.59

## References

[b1-tlsr-36-2-203] Abd Ghani MS, Abu Bakar NA, Ramadani AP, Nugraha AT, Awang K, Che Omar MT, Mohamad Taib MNA (2024). Hemisynthesis of pentacyclic triterpenoids from *Diospyros foxworthy* with *in vitro* and *in silico* antimalarial evaluation. Current Organic Chemistry.

[b2-tlsr-36-2-203] Adzu B, Amos S, Dzarma S, Muazza I, Gamaniel KS (2002). Pharmacological evidence favoring the folkloric use of *Diospyros mespiliformis* Hochst in the relief of pain and fever. Journal of Ethnopharmacology.

[b3-tlsr-36-2-203] Ahmed SA, Rahman AA, Elsayed KNM, Abd El-Mageed HR, Mohamed HS, Ahmed SA (2021). Cytotoxic activity, molecular docking, pharmacokinetic properties, and quantum mechanics calculations of the brown macroalga *Cystoseira trinodis* compounds. Journal of Biomolecular Structure and Dynamics.

[b4-tlsr-36-2-203] Aung HH, Chia LS, Goh NK, Chia TF, Ahmed AA, Pare PW, Mabry TJ (2002). Phenolic constituents from the leaves of the carnivorous plant *Nepenthes gracilis.*. Fitoterapia.

[b5-tlsr-36-2-203] Ayepola OO, Olasehinde GI, Adedeji OA, Adeyemi OO, Onile-Ere OA (2018). *In-vitro* antimicrobial activity of crude extracts of *Diospyros monbuttensis*. African Journal of Clinical and Experimental Microbiology.

[b6-tlsr-36-2-203] Bafarawa ID, Abd Ghani MS, Mohamad Taib MNA, Othman MBH, Ibrahim MN, Che Omar MT, Ramadani AP, Salsabila DN, Nugraha AT, Werdyani S, Awang K, Litaudon M (2024). Isolation, characterization, molecular docking, and antimalarial activity of chemical constituents of *Diospyros adenophora*. Malaysian Journal of Chemistry.

[b7-tlsr-36-2-203] Chaniad P, Mungthin M, Payaka A, Viriyavejakul P, Punsawad C (2021). Antimalarial properties and molecular docking analysis of compounds from *Dioscorea bulbifera* L. as new antimalarial agent candidates. BMC Complementary Medicine and Therapies.

[b8-tlsr-36-2-203] Comer E, Beaudoin JA, Kato N, Fitzgerald ME, Heidebrecht RW, Lee IVMD, Schreiber SL (2014). Diversity-oriented synthesis-facilitated medicinal chemistry: Toward the development of novel antimalarial agents. Journal of Medicinal Chemistry.

[b9-tlsr-36-2-203] da Silva GN, Maria NR, Schuck DC, Cruz LN, de Moraes MS, Nakabashi M, Graebin C, Gosmann G, Garcia CRS, Gnoatto SCB (2013). Two series of new semisynthetic triterpene derivatives: Differences in anti-malarial activity, cytotoxicity and mechanism of action. Malaria Journal.

[b10-tlsr-36-2-203] Diedrich D, Wildner AC, Silveira TF, Silva GN, Dos Santos F, da Silva EF, Gnoatto SC (2018). SERCA plays a crucial role in the toxicity of a betulinic acid derivative with potential antimalarial activity. Chemico-Biological Interactions.

[b11-tlsr-36-2-203] Dohutia C, Chetia D, Gogoi K, Sarma K (2017). Design, *in silico* and *in vitro* evaluation of curcumin analogues against *Plasmodium falciparum*. Experimental Parasitology.

[b12-tlsr-36-2-203] du Preez-Bruwer I, Mumbengegwi DR, Louw S (2022). *In vitro* antimalarial properties and chemical composition of *Diospyros chamaethamnus* extracts. South African Journal of Botany.

[b13-tlsr-36-2-203] Dzouemo LC, Mouthé Happi G, Ahmed SA, Tekapi Tsopgni WD, Nde Akuma M, Salau S, Ngeufa Happi E, Wansi JD (2022). Chemical constituents of the bark of *Zanthoxylum gilletii* (Rutaceae) and their *in vitro* antiplasmodial and molecular docking studies. Journal of Chemistry.

[b14-tlsr-36-2-203] Eberhardt J, Santos-Martins D, Tillack AF, Forli S (2021). AutoDock Vina 1.2. 0: New docking methods, expanded force field, and python bindings. Journal of Chemical Information and Modeling.

[b15-tlsr-36-2-203] Feumo Feusso H, Dieu Dongmo JD, Akak CM, Lateef M, Ahmed A, Blaise Azebaze AG, Vardamides JC (2019). Biological activities of the methanolic extracts and compounds from leaves and twigs of *Diospyros zenkeri* (Gürke) F. White (Ebenaceae). Trends in Phytochemical Research.

[b16-tlsr-36-2-203] Fotie J, Bohle DS, Leimanis ML, Georges E, Rukunga G, Nkengfack AE (2006). Lupeol long-chain fatty acid esters with antimalarial activity from *Holarrhena floribunda*. Journal of Natural Products.

[b17-tlsr-36-2-203] Ghani MSA, Zakaria N, Mohd Arshad N, Kamarulzaman EE, Awang K, Litaudon M, Taib MNA (2022). Pentacyclic triterpenoids isolated from *Diospyros foxworthyi* Bakh. (Ebenaceae) with its cytotoxic activity against HT-29 human colon cancer cell. Malaysian Journal of Chemistry.

[b18-tlsr-36-2-203] Gnoatto SC, Susplugas S, Dalla Vechia L, Ferreira TB, Dassonville-Klimpt A, Zimmer KR, Sonnet P (2008). Pharmacomodulation on the 3-acetylursolic acid skeleton: Design, synthesis, and biological evaluation of novel N-{3-[4-(3-aminopropyl) piperazinyl] propyl}-3-O-acetylursolamide derivatives as antimalarial agents. Bioorganic and Medicinal Chemistry.

[b19-tlsr-36-2-203] Gogoi K, Chetia D, Barua NC, Gogoi N, Dohutia C (2019). *In vitro* antimalarial evaluation and molecular docking analysis of Mannich base curcumin analogues against the artemisinin target *Pf*ATP6. Asian Journal of Pharmacy and Pharmacology.

[b20-tlsr-36-2-203] Kalita D, Devi N, Baishya D (2016). Foliar nutraceutical and antioxidant property of *Diospyros lanceifolia* Roxb (Ebenaceae): An important medicinal plant of Assam, India. International Advanced Research Journal in Science, Engineering and Technology.

[b21-tlsr-36-2-203] Karagöz A, Leidenberger M, Hahn F, Hampel F, Friedrich O, Marschall M, Tsogoeva SB (2019). Synthesis of new betulinic acid/betulin-derived dimers and hybrids with potent antimalarial and antiviral activities. Bioorganic and Medicinal Chemistry.

[b22-tlsr-36-2-203] Karan SK, Mondal A, Mishra SK, Pal D, Rout KK (2013). Antidiabetic effect of *Streblus asper* in streptozotocin-induced diabetic rats. Pharmaceutical Biology.

[b23-tlsr-36-2-203] Kichu M (2015). Documentation and biological and phytochemical analysis of Chungtia medicinal plants of Nagaland, India. PhD diss.

[b24-tlsr-36-2-203] Kumar S, Bhardwaj TR, Prasad DN, Singh RK (2018). Drug targets for resistant malaria: Historic to future perspectives. Biomedicine & Pharmacotherapy.

[b25-tlsr-36-2-203] Machado VR, Sandjo LP, Pinheiro GL, Moraes MH, Steindel M, Pizzolatti MG, Biavatti MW (2018). Synthesis of lupeol derivatives and their antileishmanial and antitrypanosomal activities. Natural Product Research.

[b26-tlsr-36-2-203] Malewska T (2022). Biological and phytochemical analysis of three medicinally important plants: *Prunus persica, Diospyros lanceifolia*, and *Holboellia latifolia*. PhD diss.

[b27-tlsr-36-2-203] Masia KJ, Mhlongo NN, Pooe OJ, Ibrahim MA, Kappo AP, Simelane MB (2024). Antiplasmodial potential of compounds isolated from *Ziziphus mucronata* and their binding to *Plasmodium falciparum* HGXPRT using biophysical and molecular docking studies. Naunyn-Schmiedeberg’s Archives of Pharmacology.

[b28-tlsr-36-2-203] Melariri P, Campbell W, Etusim P, Smith P (2012). *In vitro* and *in vivo* antimalarial activity of linolenic and linoleic acids and their methyl esters. Advanced Studies in Biology.

[b29-tlsr-36-2-203] Moore CM, Wang J, Lin Q, Ferreira P, Avery MA, Elokely K, Staines HM, Krishna S (2022). Selective inhibition of *Plasmodium falciparum* ATPase 6 by artemisinins and identification of new classes of inhibitors after expression in yeast. Antimicrobial Agents and Chemotherapy.

[b30-tlsr-36-2-203] Morris GM, Huey R, Lindstrom W, Sanner MF, Belew RK, Goodsell DS, Olson AJ (2009). AutoDock4 and AutoDockTools4: Automated docking with selective receptor flexibility. Journal of Computational Chemistry.

[b31-tlsr-36-2-203] Nweze C, Ibrahim H, Ndukwe GI (2019). Beta-sitosterol with antimicrobial properties from the stem bark of pomegranate (*Punica granatum* Linn). Journal of Applied Sciences and Environmental Management.

[b32-tlsr-36-2-203] Pan WH, Xu XY, Shi N, Tsang SW, Zhang HJ (2018). Antimalarial activity of plant metabolites. International Journal of Molecular Sciences.

[b33-tlsr-36-2-203] Peyrat LA, Eparvier V, Eydoux C, Guillemot JC, Stien D, Litaudon M (2016). Chemical diversity and antiviral potential in the pantropical *Diospyros* genu*s*. Fitoterapia.

[b34-tlsr-36-2-203] Plucinski MM, Talundzic E, Morton L, Dimbu PR, Macaia AP, Fortes F, Goldman I, Lucchi N, Stennies G, MacArthur JR, Udhayakumar V (2015). Efficacy of artemether-lumefantrine and dihydroartemisinin-piperaquine for treatment of uncomplicated malaria in children in Zaire and Uíge provinces, Angola. Antimicrobial Agents and Chemotherapy.

[b35-tlsr-36-2-203] POWO (2025). Diospyros L: Plants of the World Online: Kew Science.

[b36-tlsr-36-2-203] Rathore K, Singh VK, Jain P, Rao SP, Ahmed Z, Singh VD (2014). *In vitro* and *in vivo* antiadipogenic, hypolipidemic, and antidiabetic activity of *Diospyros melanoxylon* (Roxb). Journal of Ethnopharmacology.

[b37-tlsr-36-2-203] Rauf A, Uddin G, Siddiqui BS, Khan H (2015). *In vivo* sedative and muscle relaxants activity of *Diospyros lotus* L. Asian Pacific Journal of Tropical Biomedicine.

[b38-tlsr-36-2-203] Rauf A, Uysal S, Hadda TB, Uddin G, Nawaz MA, Khan H, Siddiqui BS, Raza M, Bawazeer S, Zengin G (2017). *In vivo* and *in silico* sedative-hypnotic like activity of 7-methyl juglone isolated from *Diospyros lotus* L. Biomedicine and Pharmacotherapy.

[b39-tlsr-36-2-203] Ruphin FP, Baholy R, Emmanuel R, Amelie R, Martin MT, Koto-te-Nyiwa N (2014). Isolation and structural elucidation of cytotoxic compounds from the root bark of *Diospyros quercina* (Baill.) endemic to Madagascar. Asian Pacific Journal of Tropical Biomedicine.

[b40-tlsr-36-2-203] Salas-Burgos A, Iserovich P, Zuniga F, Vera JC, Fischbarg J (2004). Predicting the three-dimensional structure of the human facilitative glucose transporter glut1 by a novel evolutionary homology strategy: Insights on the molecular mechanism of substrate migration, and binding sites for glucose and inhibitory molecules. Biophysical Journal.

[b41-tlsr-36-2-203] Shibeshi MA, Kifle ZD, Atnafie SA (2020). Antimalarial drug resistance and novel targets for antimalarial drug discovery. Infection and Drug Resistance.

[b42-tlsr-36-2-203] Sikam KG, Happi GM, Ahmed SA, Wakeu BNK, Meikeu LZ, Salau S, Wansi JD (2022). *In vitro* antiplasmodial, molecular docking and pharmacokinetics studies of specialized metabolites from *Tetrapleura tetraptera* (Fabaceae). South African Journal of Botany.

[b43-tlsr-36-2-203] Singh A, Mukhtar HM, Kaur H, Kaur L (2020). Investigation of antiplasmodial efficacy of lupeol and ursolic acid isolated from *Ficus benjamina* leaves extract. Natural Product Research.

[b44-tlsr-36-2-203] Smilkstein M, Sriwilaijaroen N, Kelly JX, Wilairat P, Riscoe M (2004). Simple and inexpensive fluorescence-based technique for high-throughput antimalarial drug screening. Antimicrobial Agents and Chemotherapy.

[b45-tlsr-36-2-203] Takahashi O, Masuda Y, Muroya A, Furuya T (2010). Theory of docking scores and its application to a customizable scoring function. SAR and QSAR in Environmental Research.

[b46-tlsr-36-2-203] Tameye NSJ, Akak CM, Tabekoueng GB, Mkounga P, Bitchagno GTM, Lenta BN, Nkengfack AE (2022). Chemical constituents from *Diospyros fragrans* Gürke (Ebenaceae). Biochemical Systematics and Ecology.

[b47-tlsr-36-2-203] Tangmouo JG, Ho R, Matheeussen A, Lannang AM, Komguem J, Messi BB, Maes L, Hostettmann K (2010). Antimalarial activity of extract and norbergenin derivatives from the stem bark of *Diospyros sanza-minika* A. Chevalier (Ebenaceae). Phytotherapy Research.

[b48-tlsr-36-2-203] Thing T, Yean Shan L, Siow Ping T, Awang K, Nafiah MA, Ahmad K (2014). Phytochemical study of stem bark from *Alstonia spathulata* Bl. (Apocynaceae). EDUCATUM: Journal of Science, Mathematics, and Technology.

[b49-tlsr-36-2-203] Trager W, Jensen J (1976). Human malaria parasites in continuous culture. Science.

[b50-tlsr-36-2-203] Trott O, Olson AJ (2010). AutoDock Vina: Improving the speed and accuracy of docking with a new scoring function, efficient optimization, and multithreading. Journal of Computational Chemistry.

[b51-tlsr-36-2-203] Tsamesidis I, Mousavizadeh F, Egwu CO, Amanatidou D, Pantaleo A, Benoit-Vical F, Giannis A (2022). *In vitro* and *in silico* antimalarial evaluation of FM-AZ, a new artemisinin derivative. Medicines.

[b52-tlsr-36-2-203] Wisetsai A, Schevenels FT, Lekphrom R (2021). Chemical constituents and their biological activities from the roots of *Diospyros filipendula*. Natural Product Research.

[b53-tlsr-36-2-203] World Health Organization (2024). World malaria report 2024.

[b54-tlsr-36-2-203] Yang J, He Y, Li Y, Zhang X, Wong YK, Shen S, Wang J (2020). Advances in the research on the targets of anti-malaria actions of artemisinin. Pharmacology and Therapeutics.

[b55-tlsr-36-2-203] Yusuf AJ, Abdullahi MI, Nasir I, Yunusa A, Alebiosu CO, Muhammad AA (2023). Isolation and characterization of prophylactic antimalarial agents from *Ochna kibbiensis* leaves. Drugs and Drug Candidates.

