# Supplementary material for: In vitro Antiplasmodial and Molecular Docking Studies of Chemical Constituent Isolated from the Bark of Diospyros lanceifolia (Ebenaceae)
Source: Trop Life Sci Res. 2025 Jul 31;36(2):203–27. doi: 10.21315/tlsr2025.36.2.10 (PMC12618094; doi:10.21315/tlsr2025.36.2.10)
Supplement: Supplementary file 1 [file TLSR-36-2-203-supplementary.pdf]

SUPPLEMENTARY MATERIALS

In vitro Antiplasmodial and Molecular Docking Studies of Chemical Constituent Isolated from the Bark of *Diospyros lanceifolia* (Ebenaceae)

[1,2]Ibrahim Dankane Bafarawa, [1]Muhammad Solehin Abd Ghani, [3]Arba Pramundita Ramadani, [3]Shofiatul Fuadah, [3]Sista Werdyani, [4]Unang Supratman, [1]Muhammad Bisyrul Hafi Othman, [1]Mohamad Nasir Mohamad Ibrahim, [5]Khalijah Awang, [6]Marc Litaudon, [7]Mohammad Tasyriq Che Omar, [8]Habibah A. Wahab, and [1]Mohamad Nurul Azmi[*]

[1]School of Chemical Sciences, Universiti Sains Malaysia, 11800 Minden, Penang, Malaysia

[2]Umaru Ali Shinkafi Polytechnic Sokoto, P.M.B. 2356 Sokoto State, Nigeria

[3]Department of Pharmacy, Universitas Islam Indonesia, Jl. Kaliurang KM 14.4 Sleman, Yogyakarta 55584, Indonesia

[4]Department of Chemistry, Faculty of Mathematics and Natural Sciences, Universitas Padjadjaran, 45363 Jatinangor, Indonesia

[5]Department of Chemistry, Faculty of Science, University of Malaya, 50603 Kuala Lumpur, Malaysia

[6]Institute de Chimie des Substances Naturelles, CNRS-ICSN UPR2301, Univ. Paris-Sud 11, av. de la Terrasse, 91198 Gif-sur-Yvette, France

[7]Biological Section, School of Distance Education, Universiti Sains Malaysia, 11800 Minden, Penang, Malaysia

[8]School of Pharmaceutical Science, Universiti Sains Malaysia, 11800 Minden, Penang, Malaysia

*Correspondence: mnazmi@usm.my

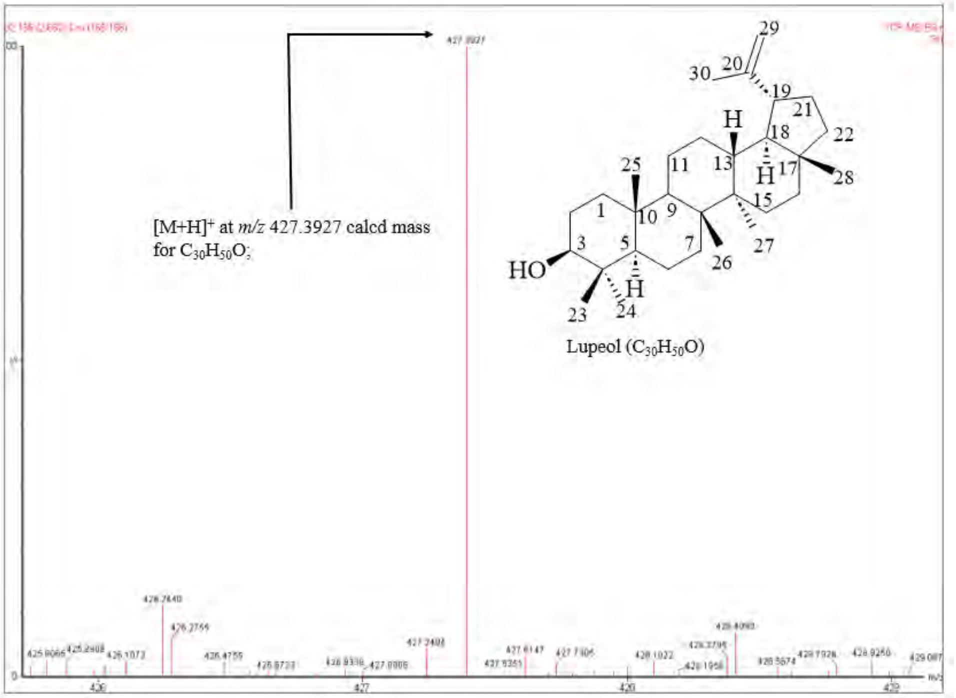

Figure S1: HRMS spectrum of **1**

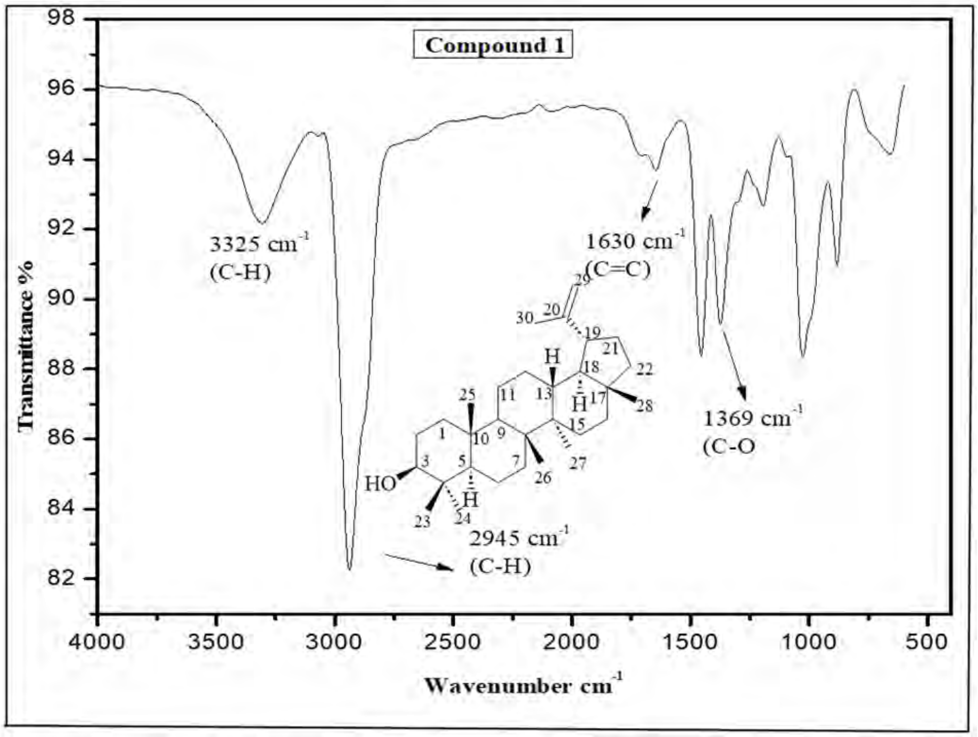

Figure S2: FT-IR spectrum of **1**

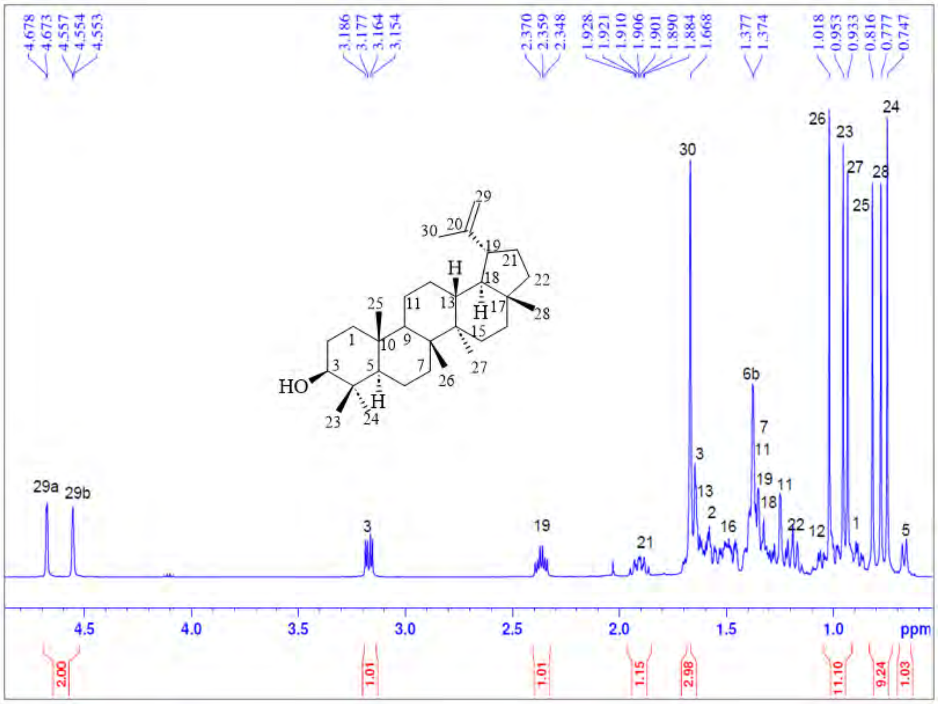

Figure S3: ^{1}H-NMR (500 MHz, $CDCl_3$) spectrum of **1**

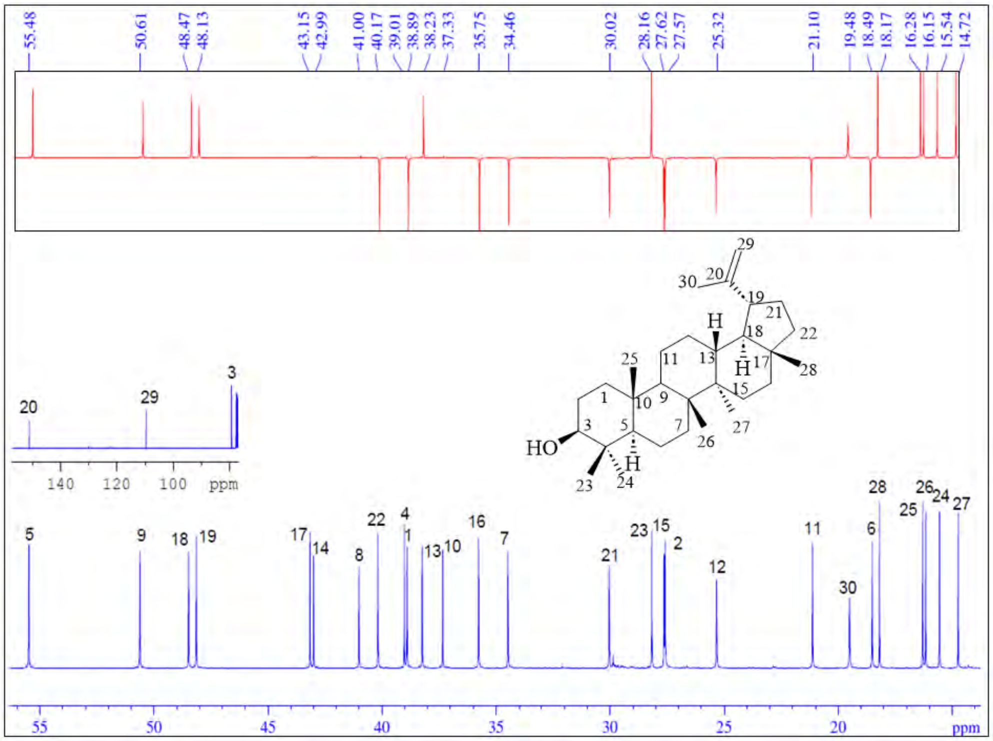

Figure S4: ^{13}C-NMR and DEPT-135 (125 MHz, $CDCl_3$) spectrum of **1**

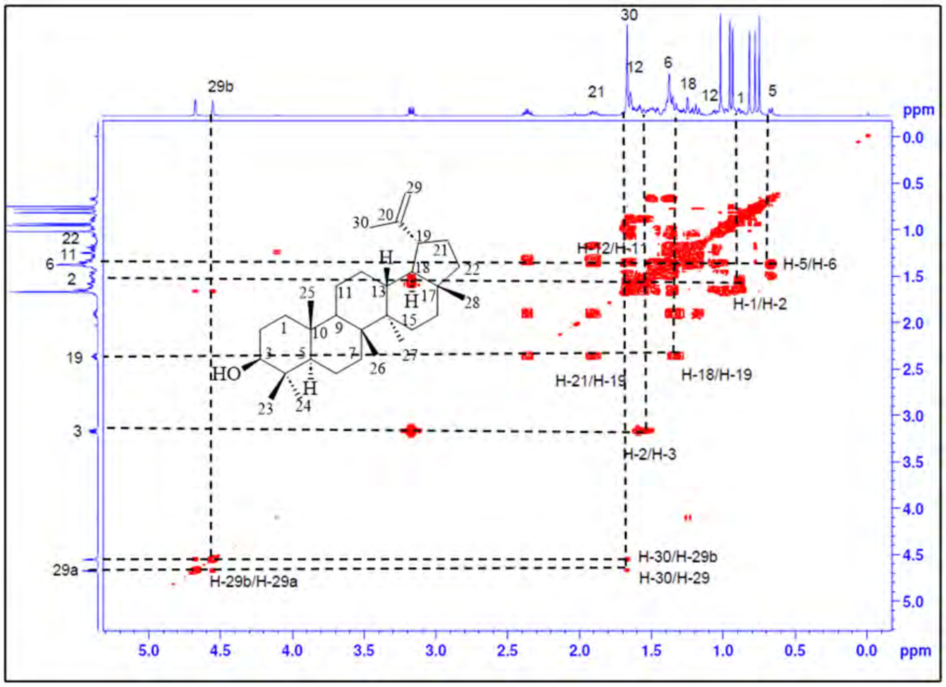

Figure S5: ^{1}H-^{1}H COSY spectrum of **1**

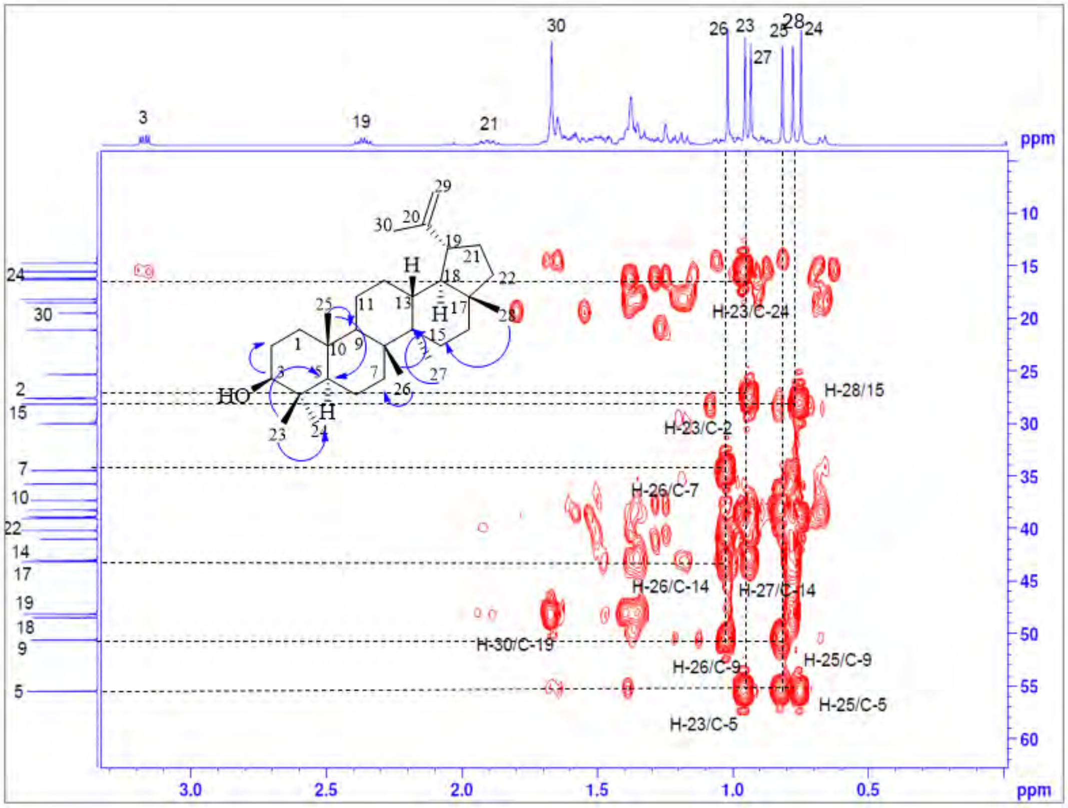

Figure S6: HMBC spectrum of **1**

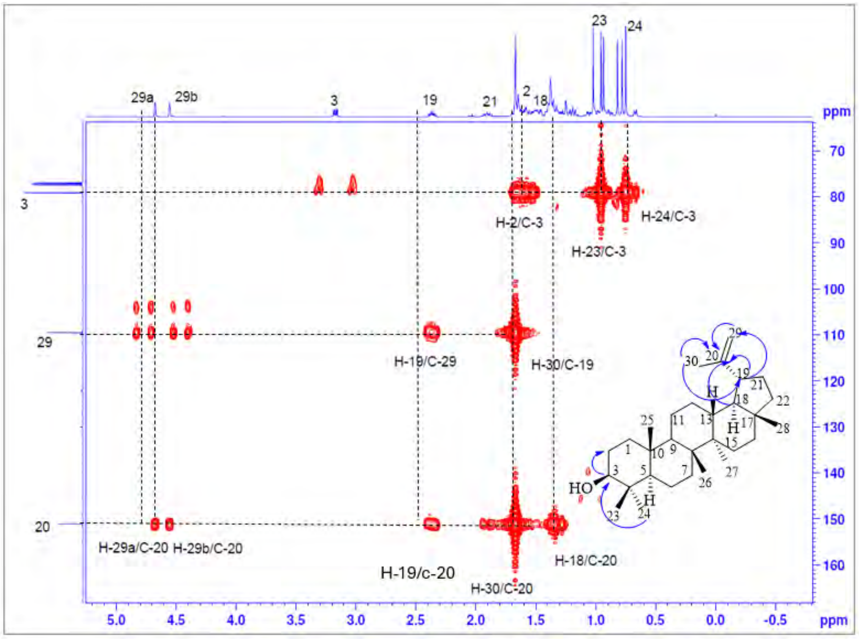

Figure S7: HMBC spectrum of **1** (expanded)

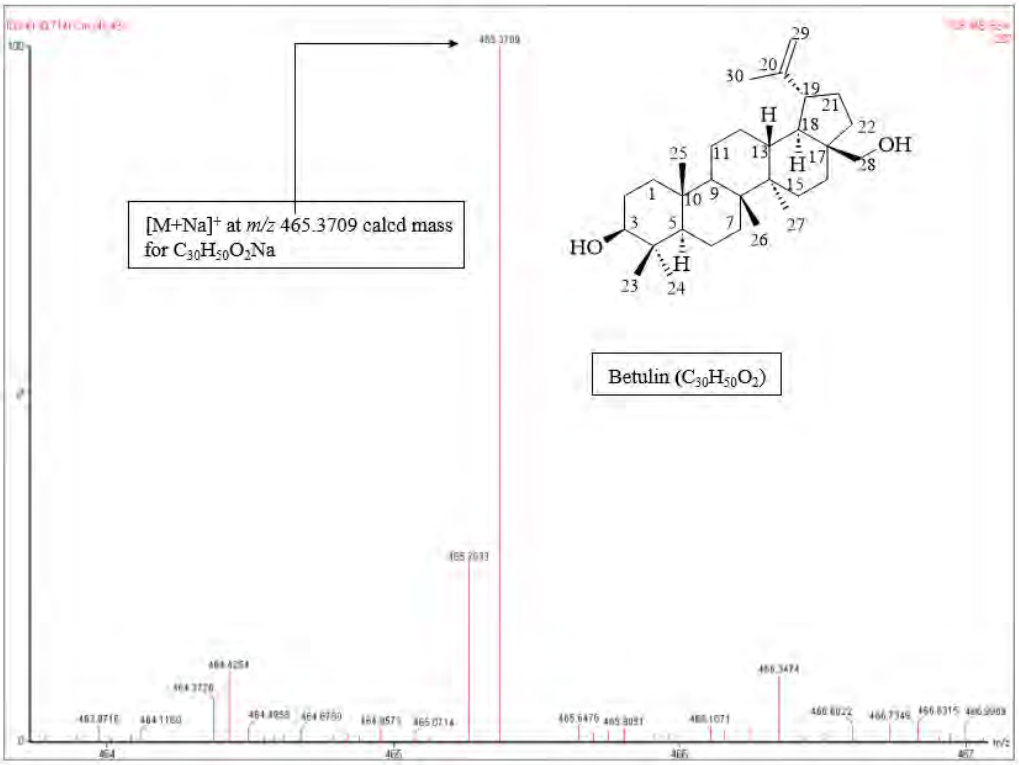

Figure S8: HRMS spectrum of **2**

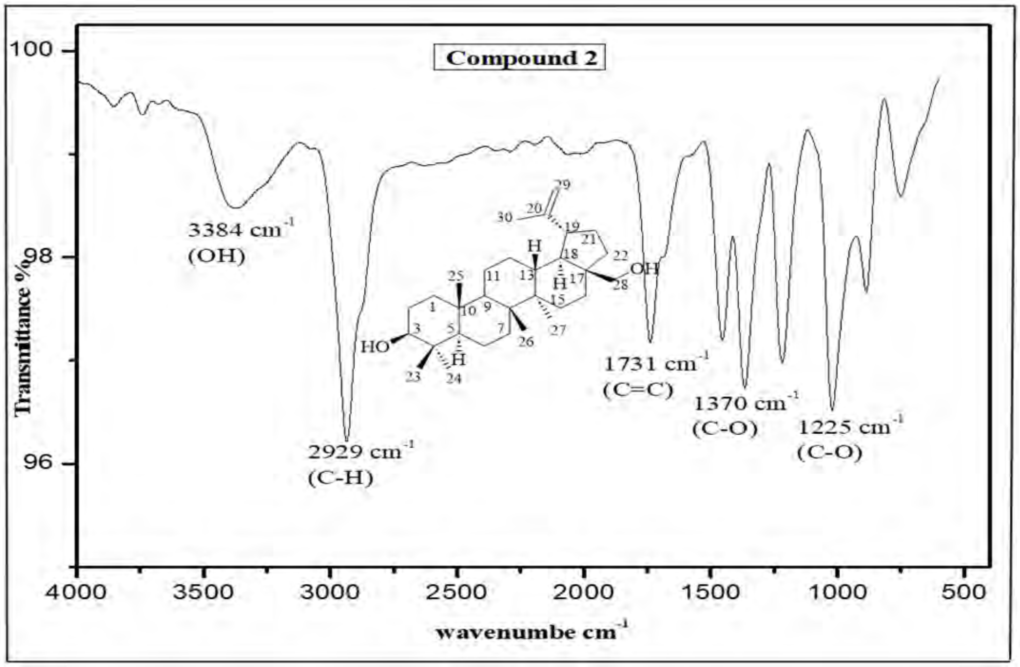

Figure S9: FT-IR spectrum of **2**

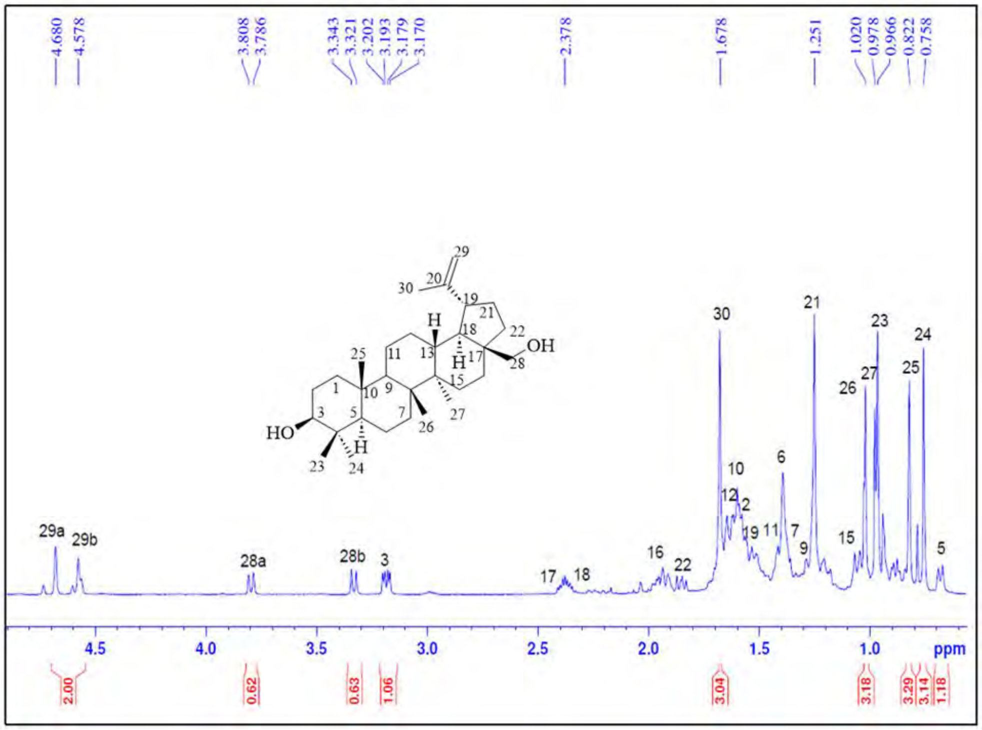

Figure S10: ^{1}H-NMR (500 MHz, $CDCl_3$) spectrum of **2**

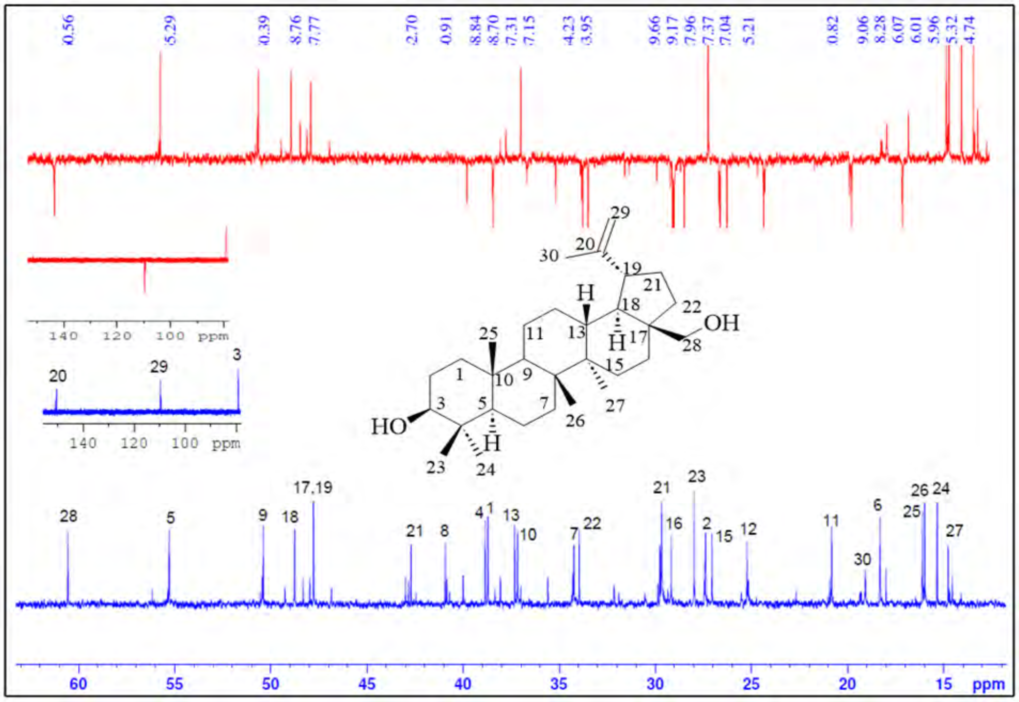

Figure S11: ^{13}C-NMR and DEPT-135 (125 MHz, $CDCl_3$) spectrum of **2**

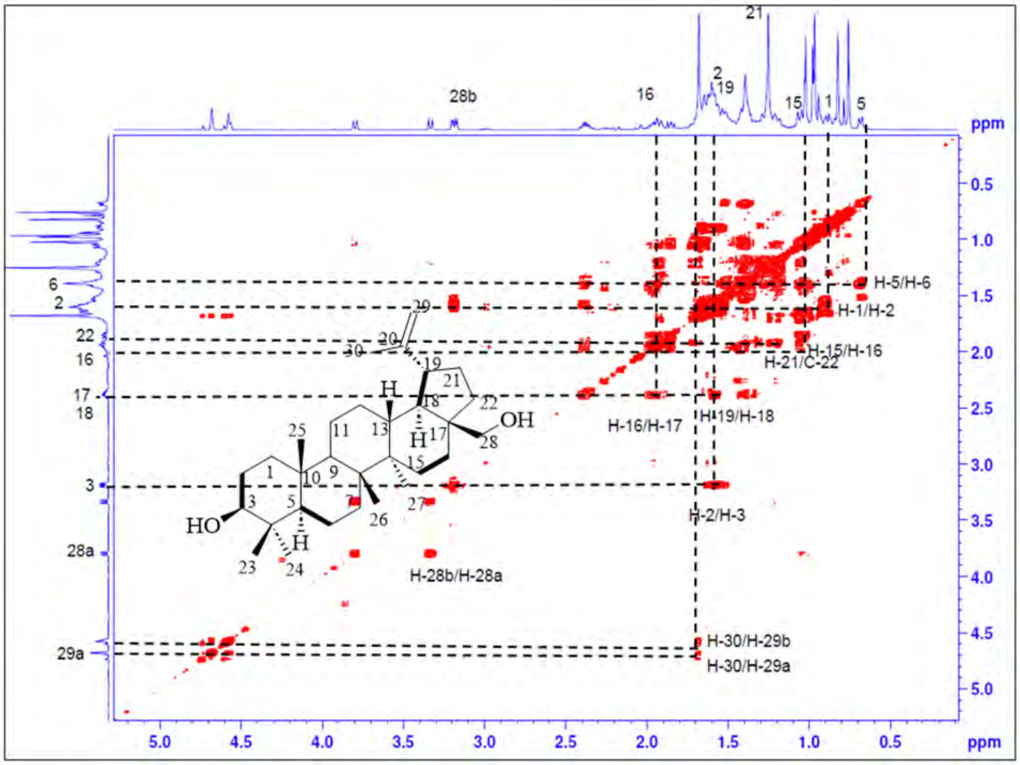

Figure S12: ^{1}H-^{1}H COSY spectrum of **2**

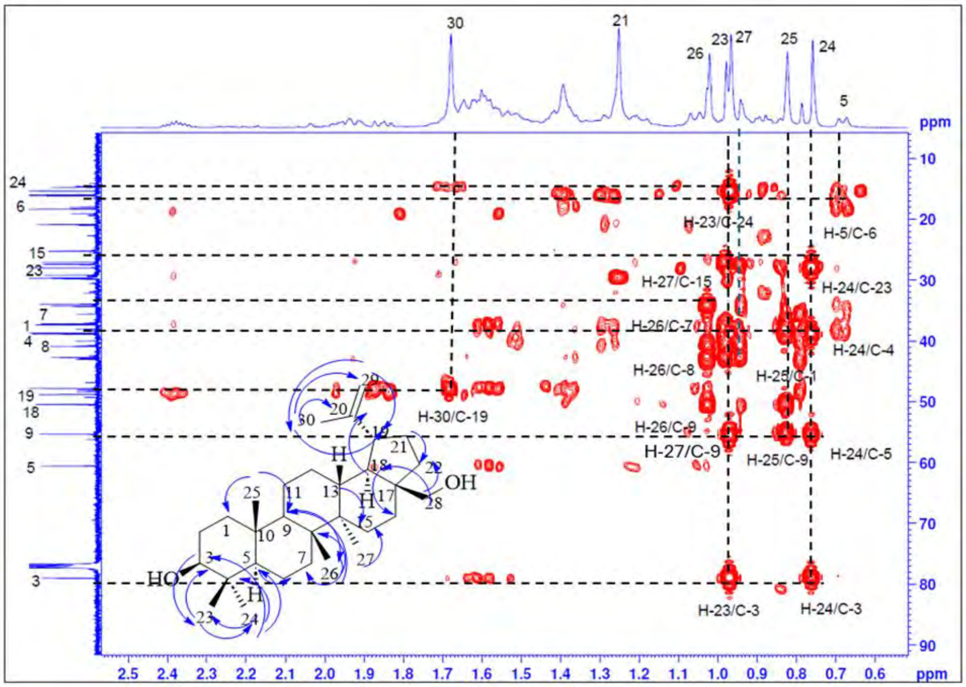

Figure S13: HMBC spectrum of **2**

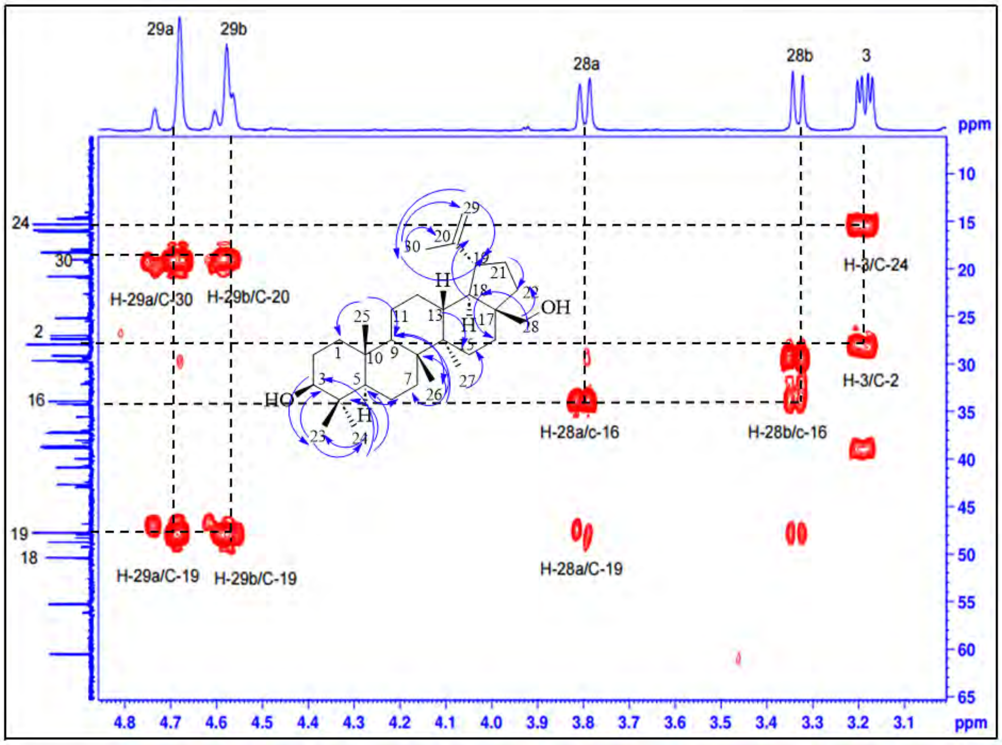

Figure S14: HMBC spectrum of **2** (Expanded)

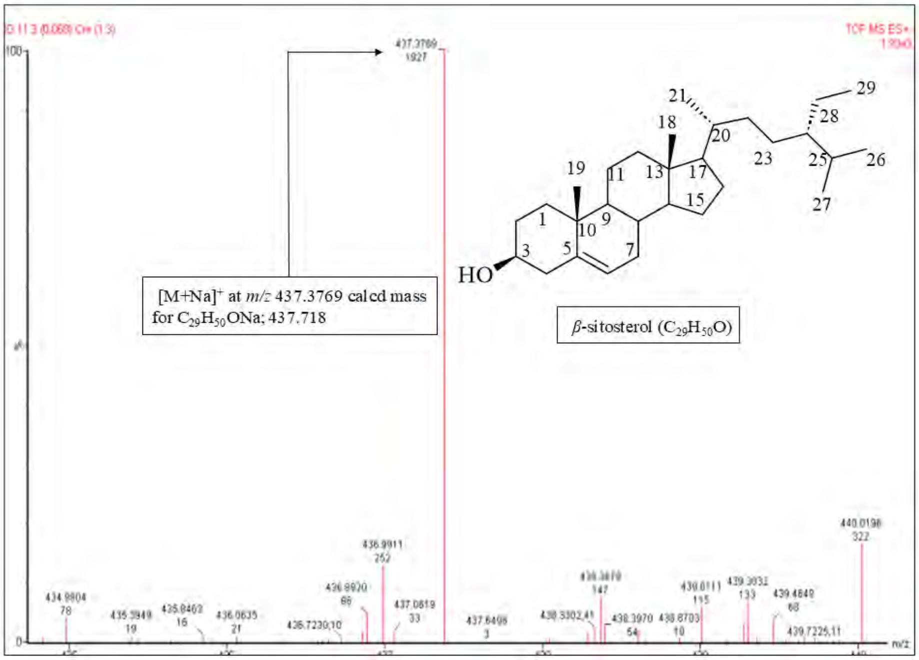

Figure S15: HRMS spectrum of **3**

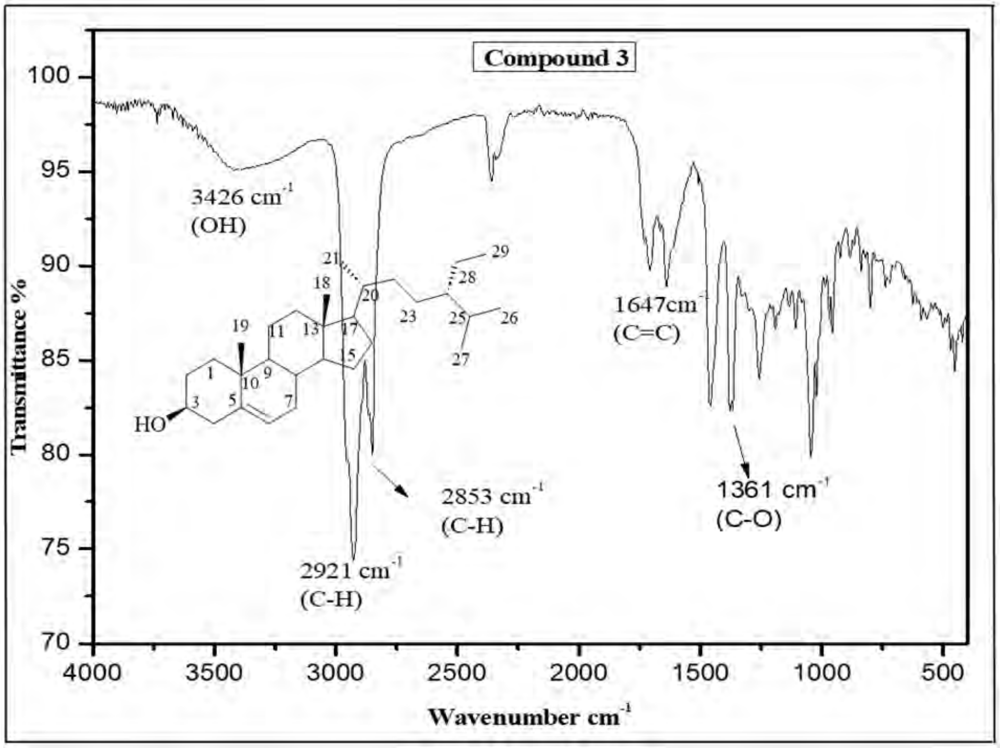

Figure S16: FT-IR spectrum of **3**

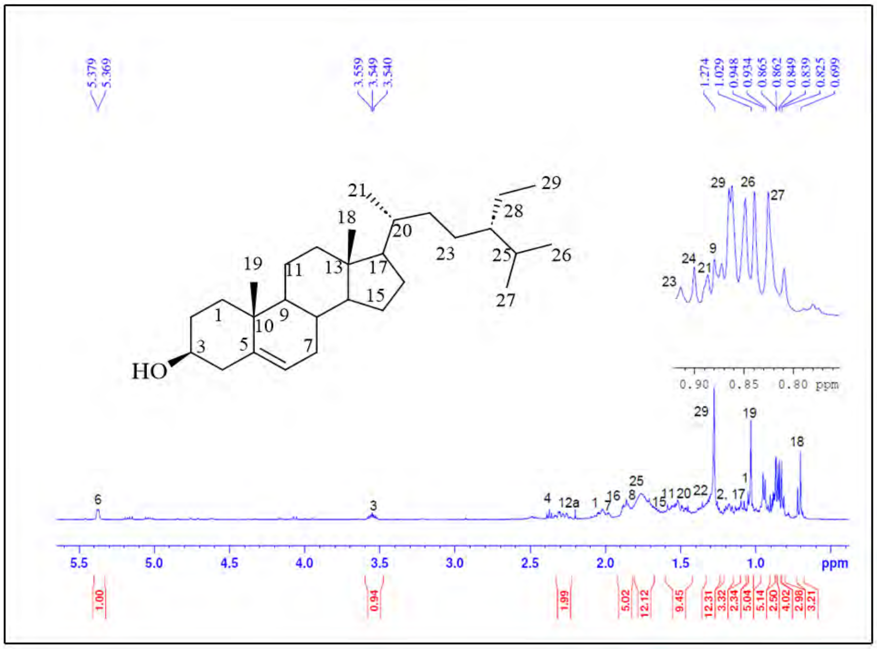

Figure S17: ^{1}H-NMR (500 MHz in $CDCl_3$) spectrum of **3**

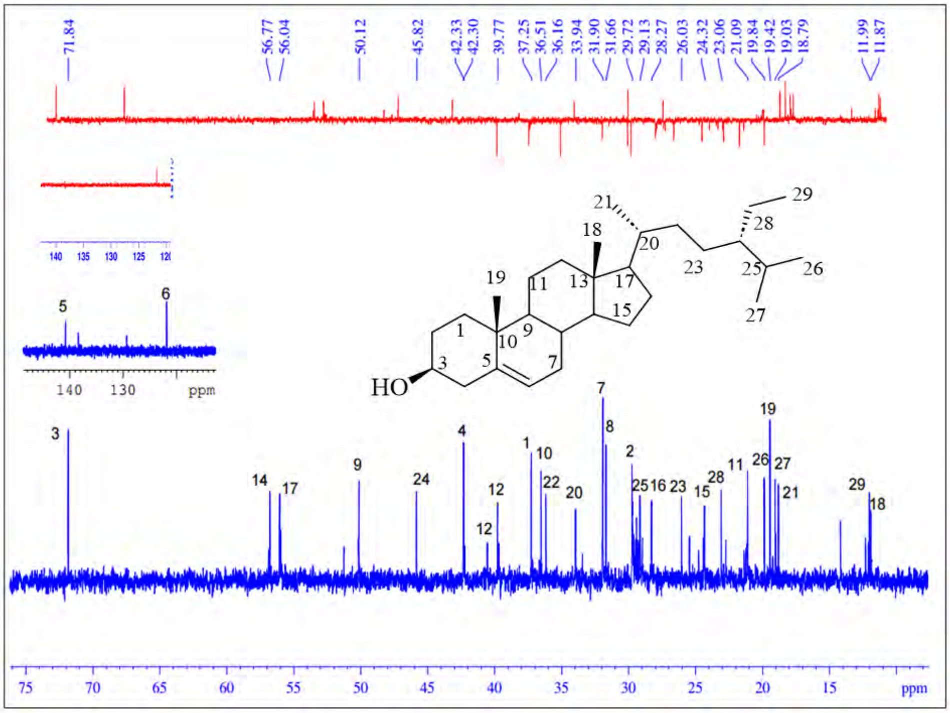

Figure S18: ^{13}C-NMR and DEPT-135 (125 MHz in $CDCl_3$) spectrum of **3**

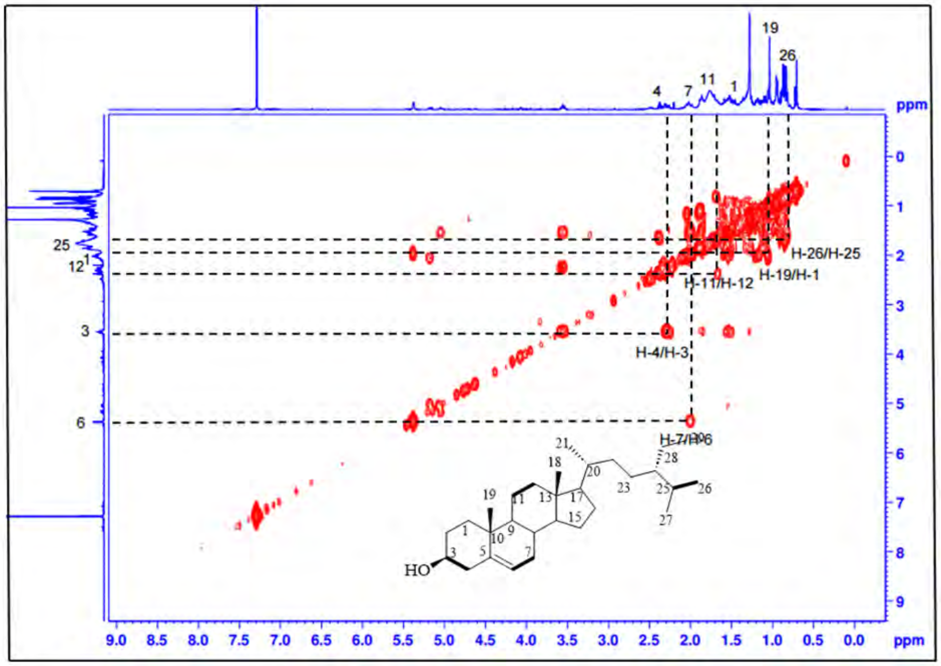

Figure S19: ^{1}H-^{1}H COSY spectrum of **3**

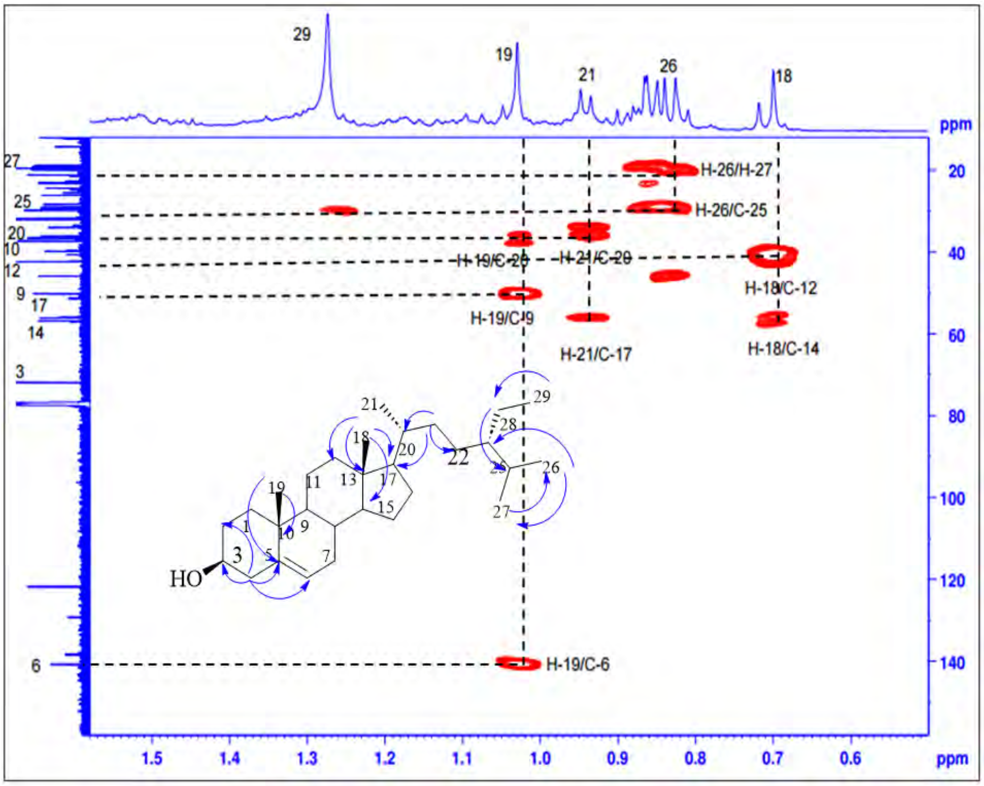

Figure S20: HMBC spectrum of **3**

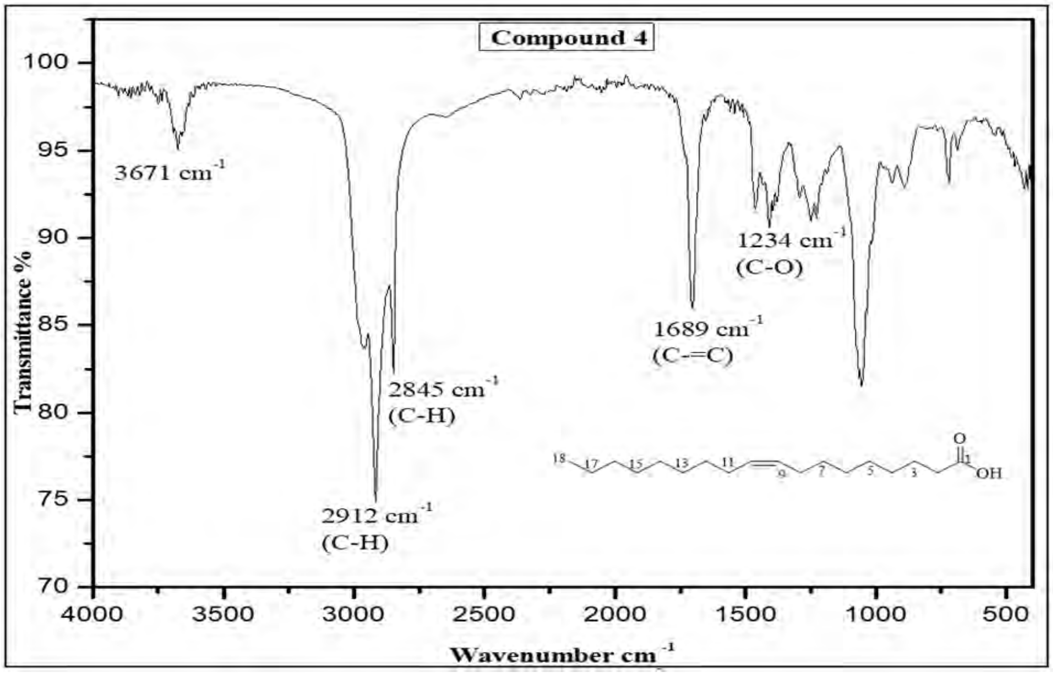

Figure S21: FT-IR spectrum of **4**

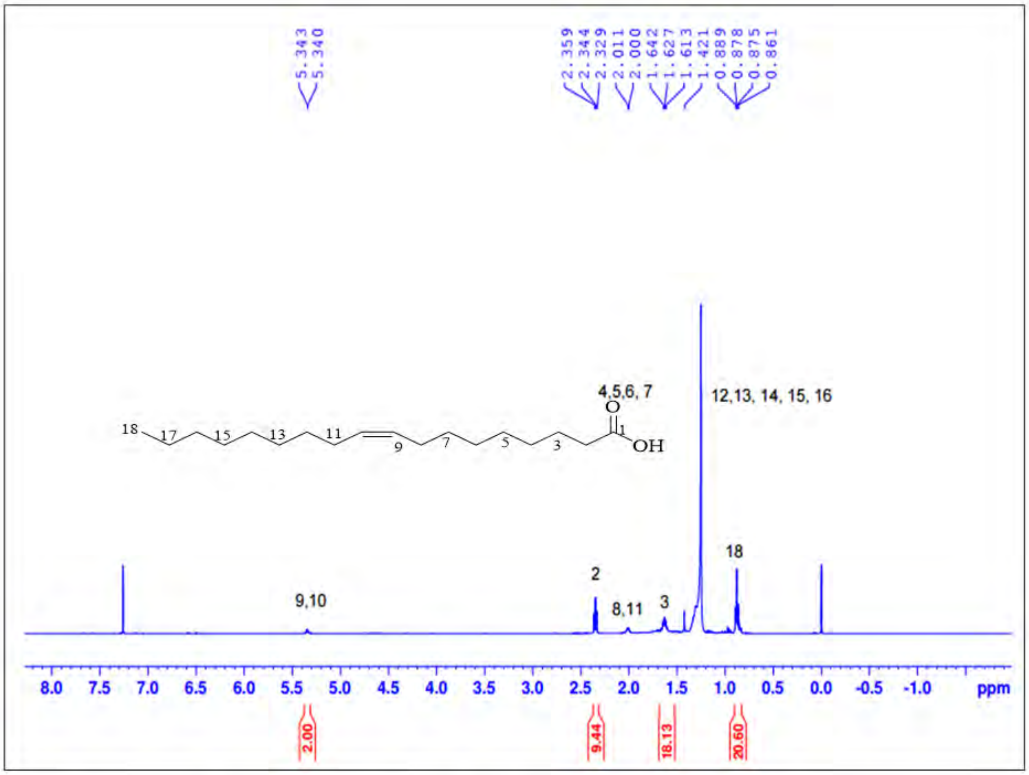

Figure S22: ^{1}H-NMR (500 MHz in $CDCl_3$) spectrum of **4**

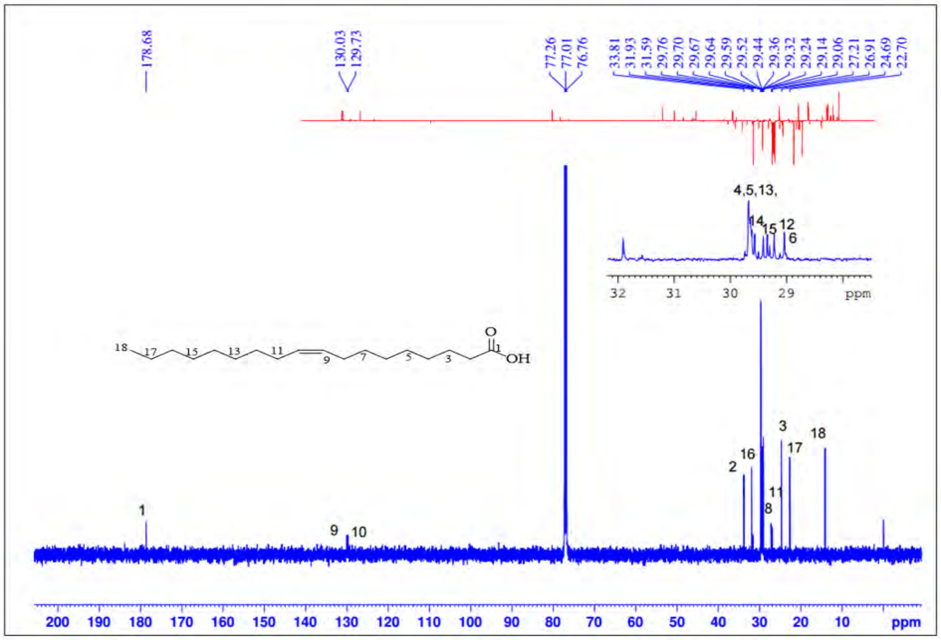

Figure S23: ^{13}C-NMR and DEPT-135 (125 MHz in $CDCl_3$) spectrum of **4**

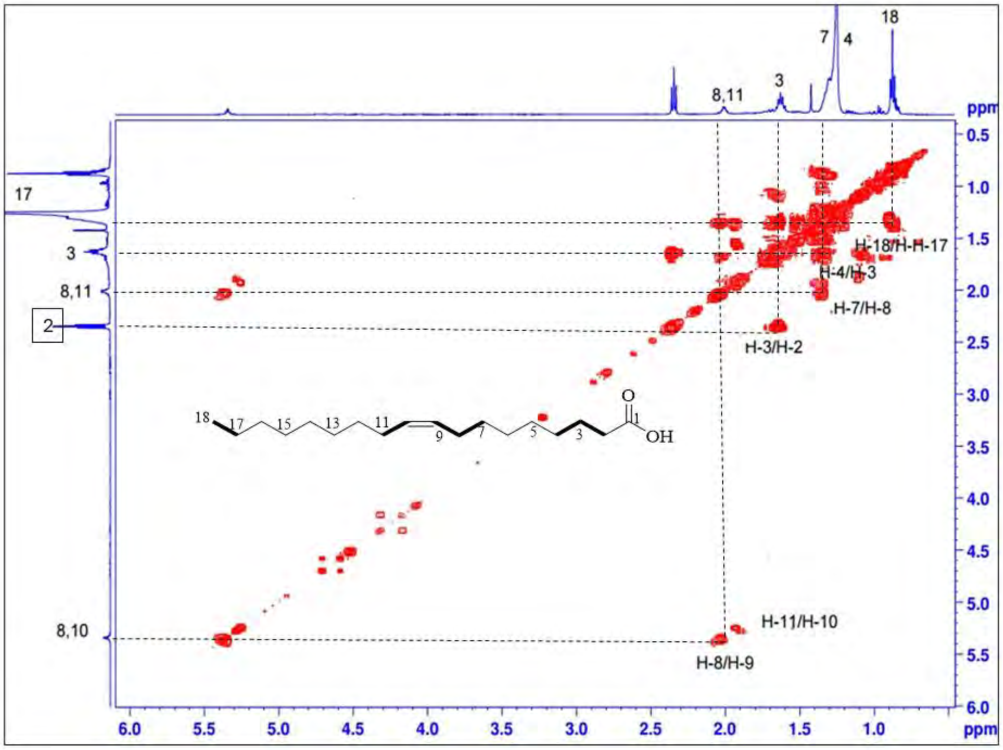

Figure S24: ^{1}H-^{1}H COSY spectrum of **4**

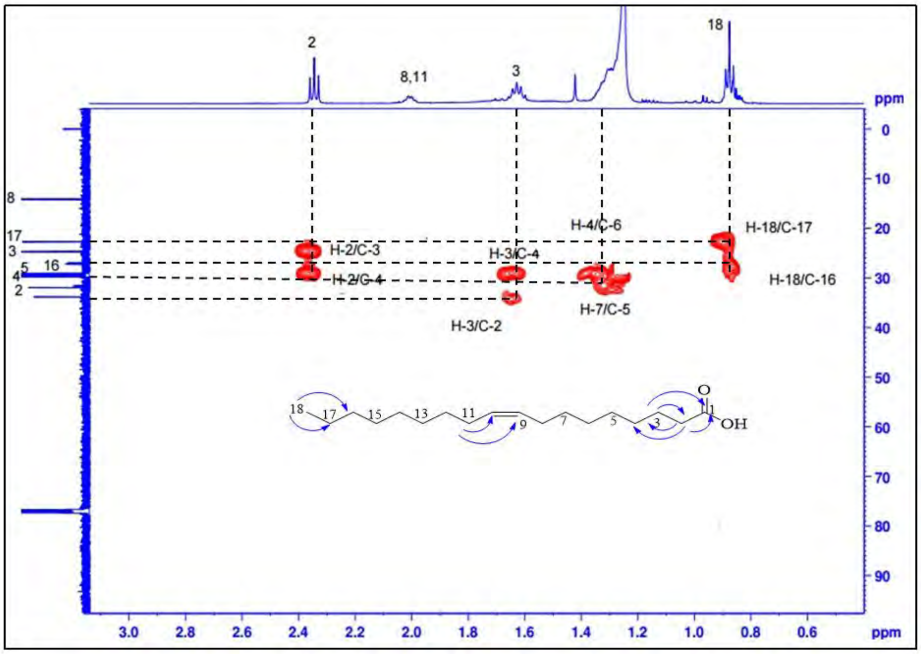

Figure S25: HMBC spectrum of **4**

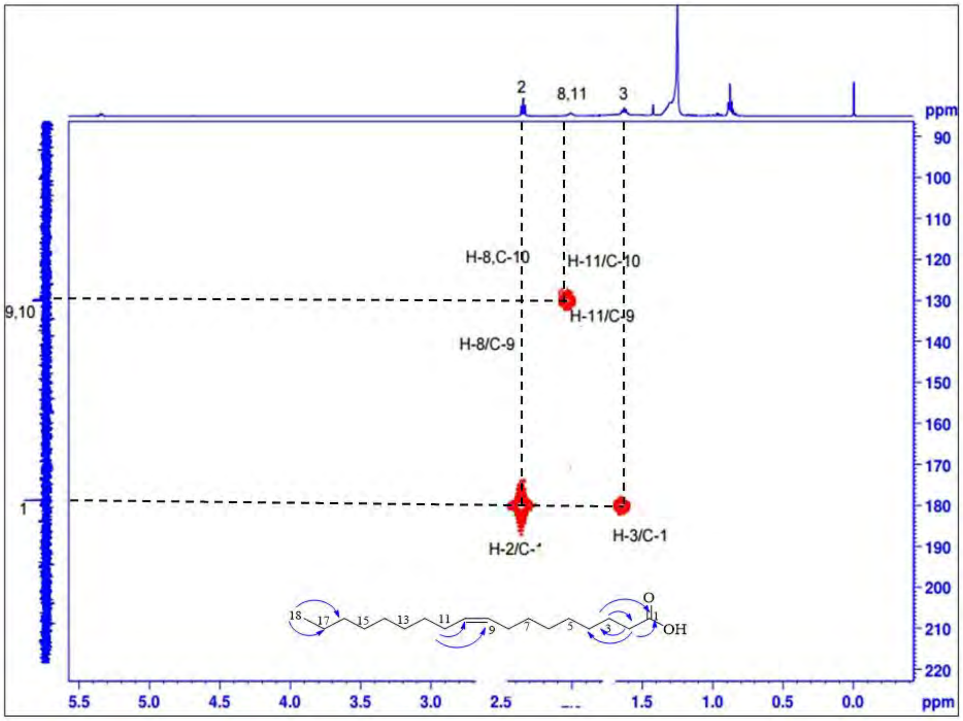

Figure S26: HMBC spectrum of **4** (Expanded)

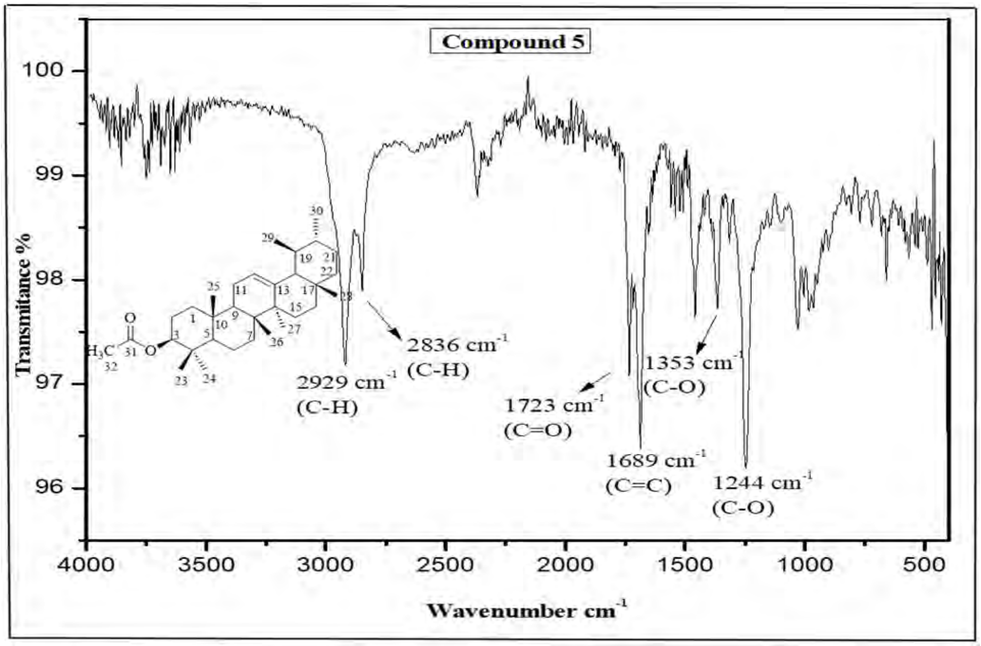

Figure S27: FT-IR spectrum of **5**

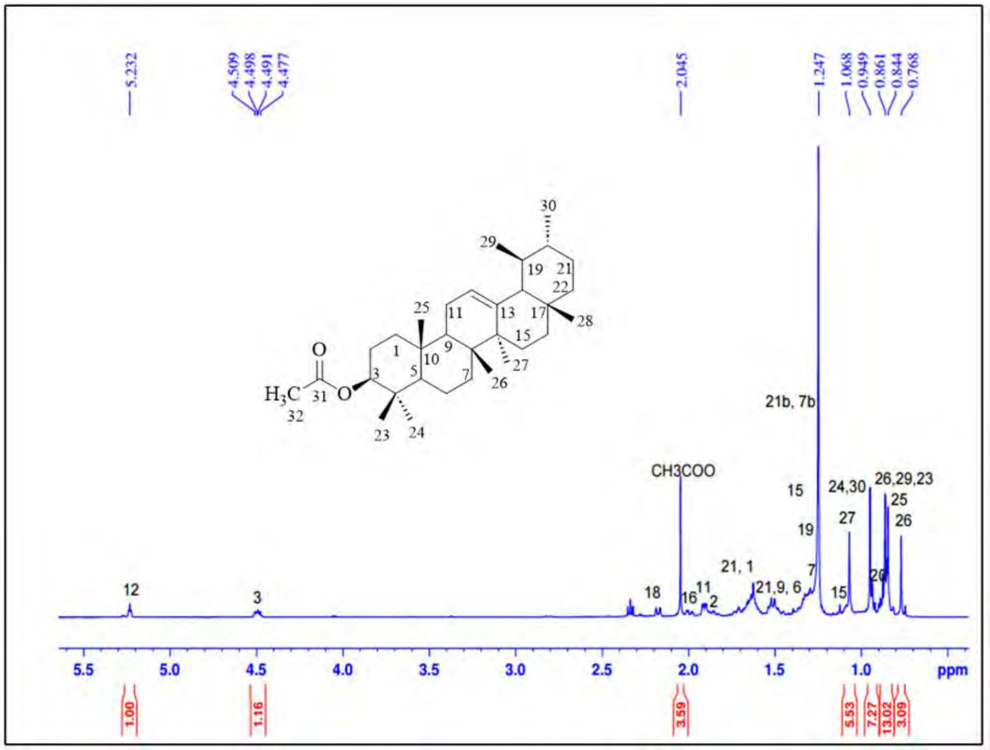

Figure S28: ^{1}H-NMR spectrum of compound **5**

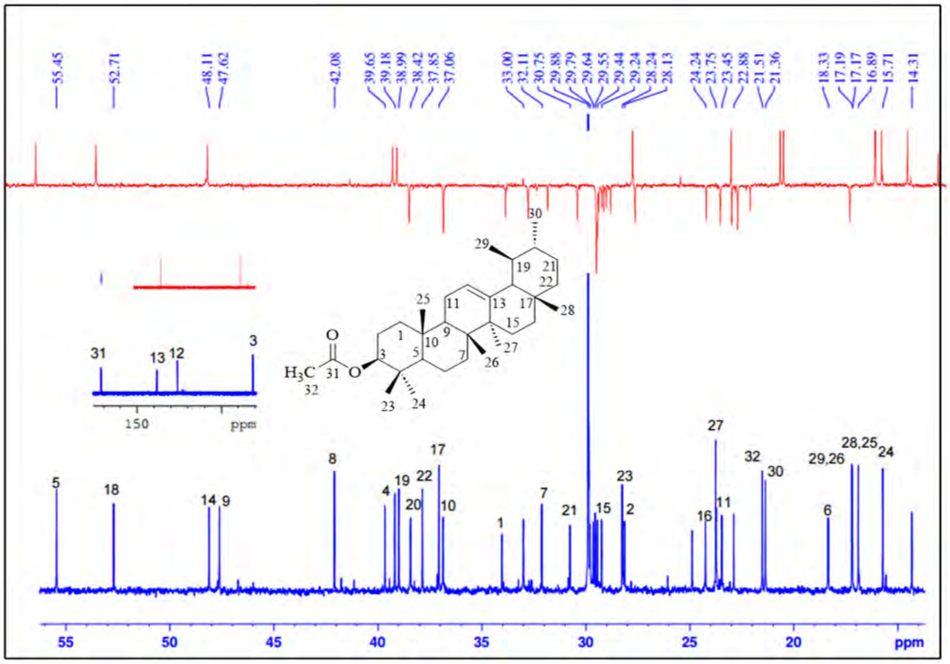

Figure S29: ^{13}C-NMR and DEPT-135 (125 MHz in $CDCl_3$) spectrum of **5**

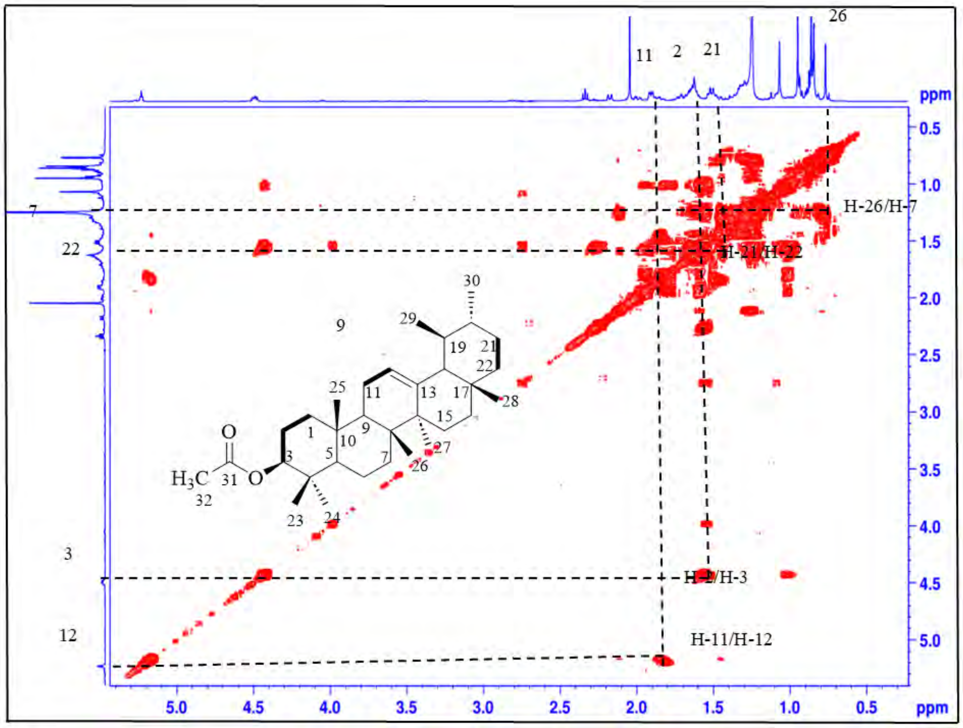

Figure S30: ^{1}H-^{1}H COSY spectrum of **5**

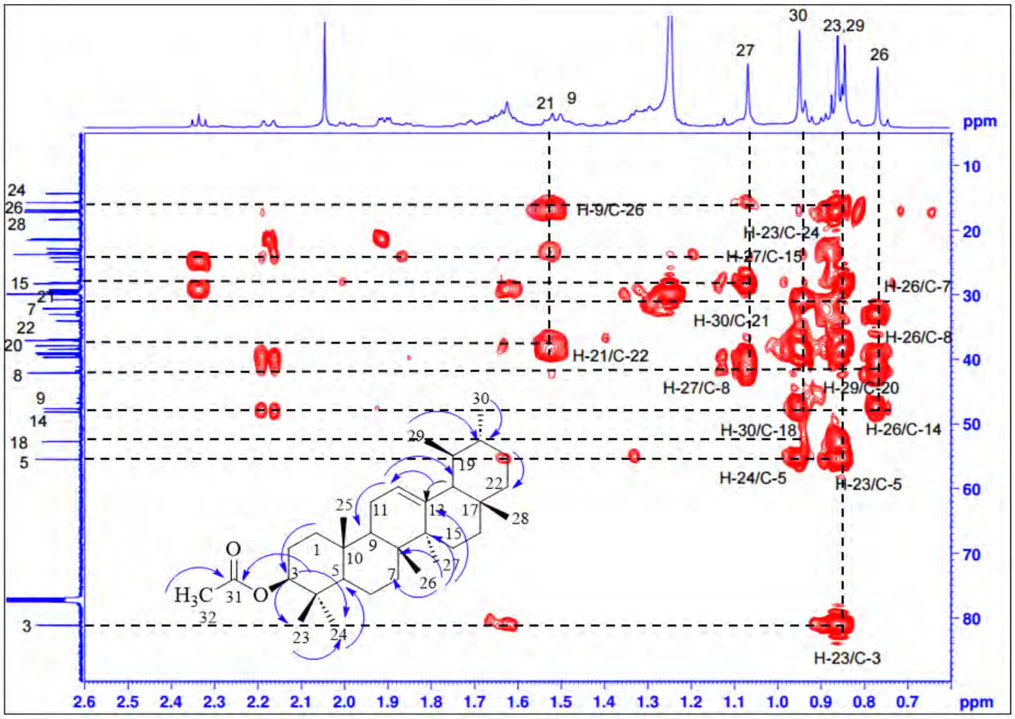

Figure S31: HMBC spectrum of compound **5**

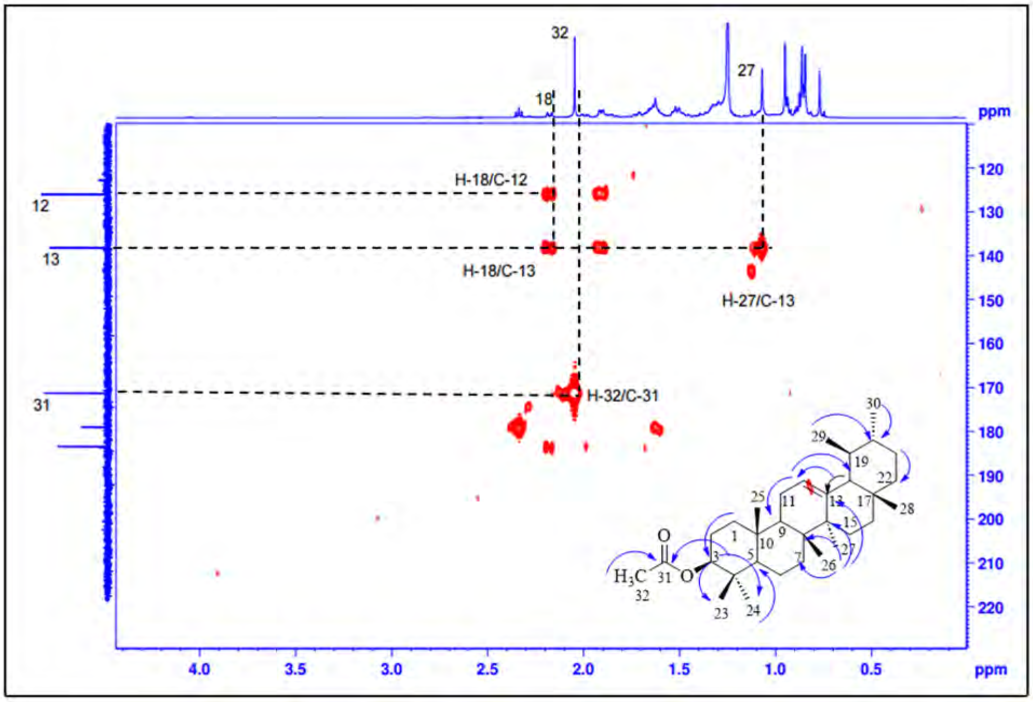

Figure S32: HMBC spectrum of **5**

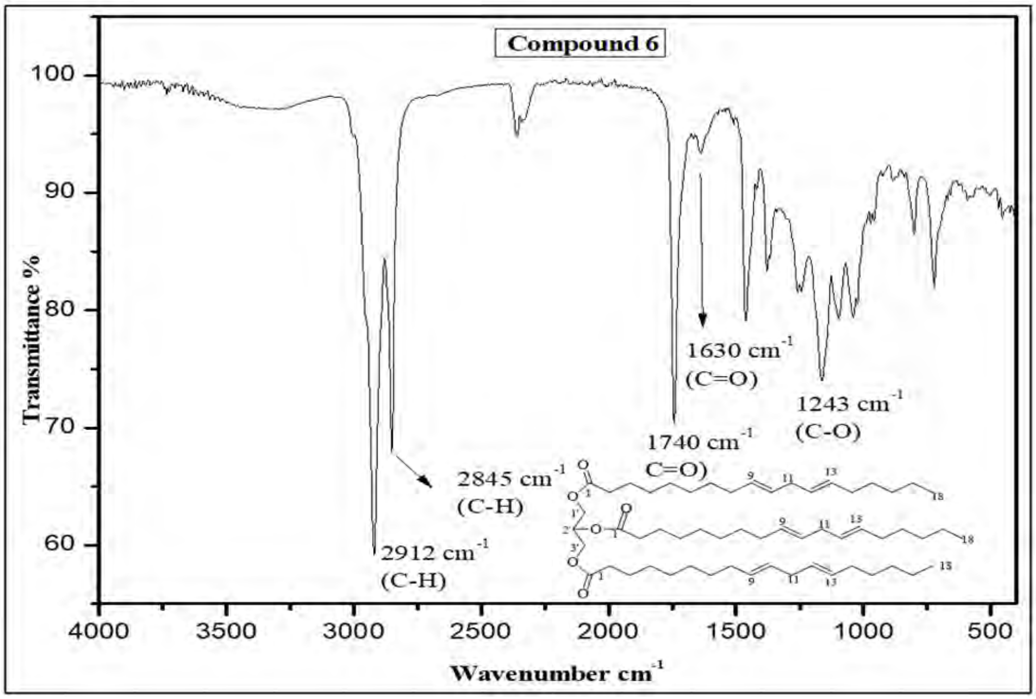

Figure S33: FT-IR spectrum of **6**

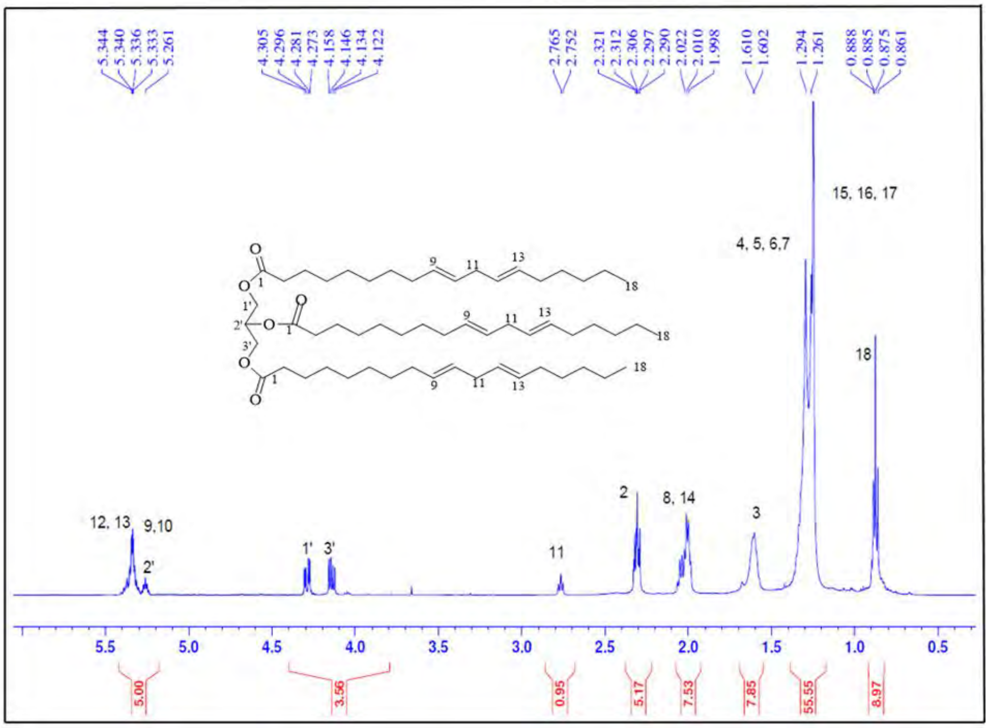

Figure S34: ^{1}H-HNMR spectrum of **6**

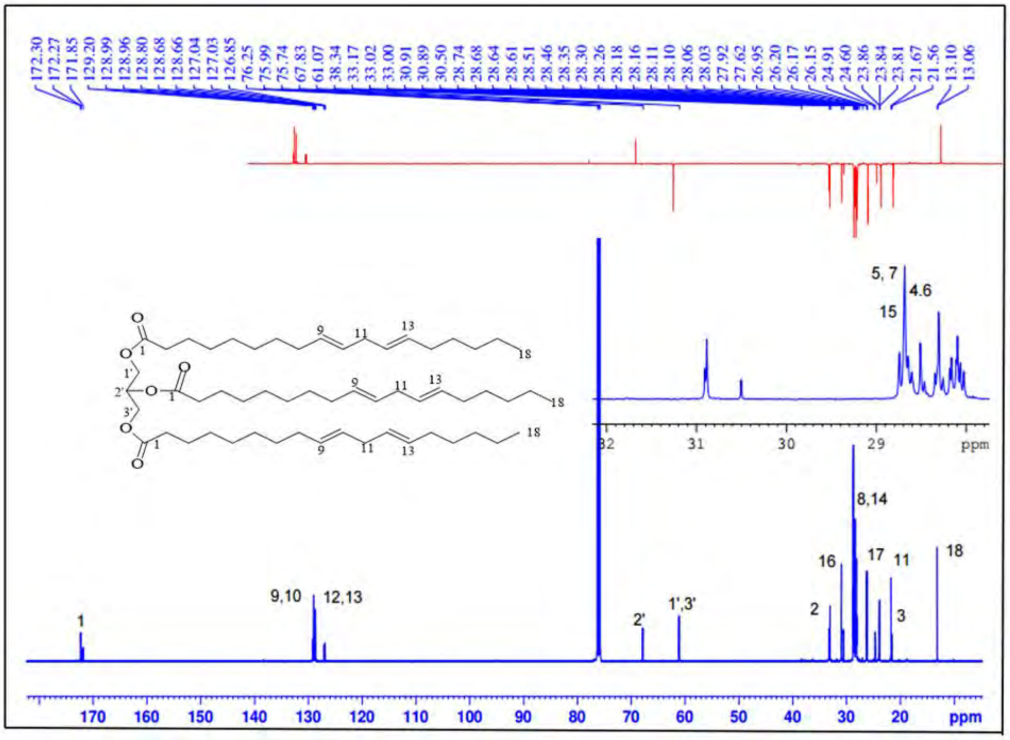

Figure S35: ^{13}C-NMR and DEPT-135 (125 MHz in $CDCl_3$) spectrum of **6**

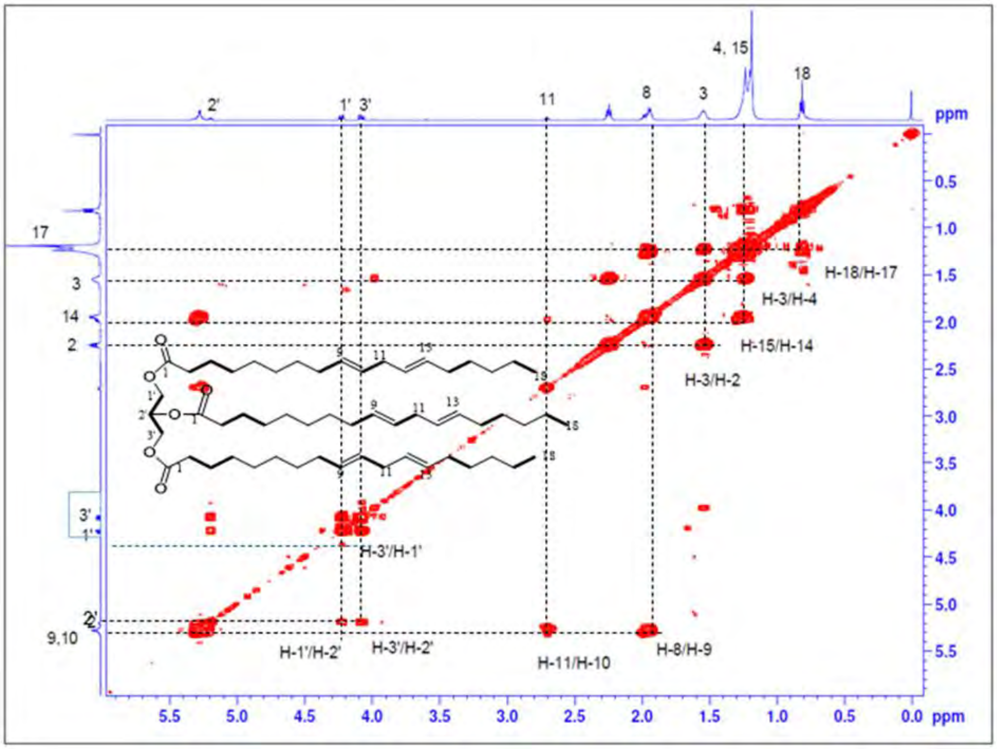

Figure S36: ^{1}H-^{1}H COSY spectrum of **6**

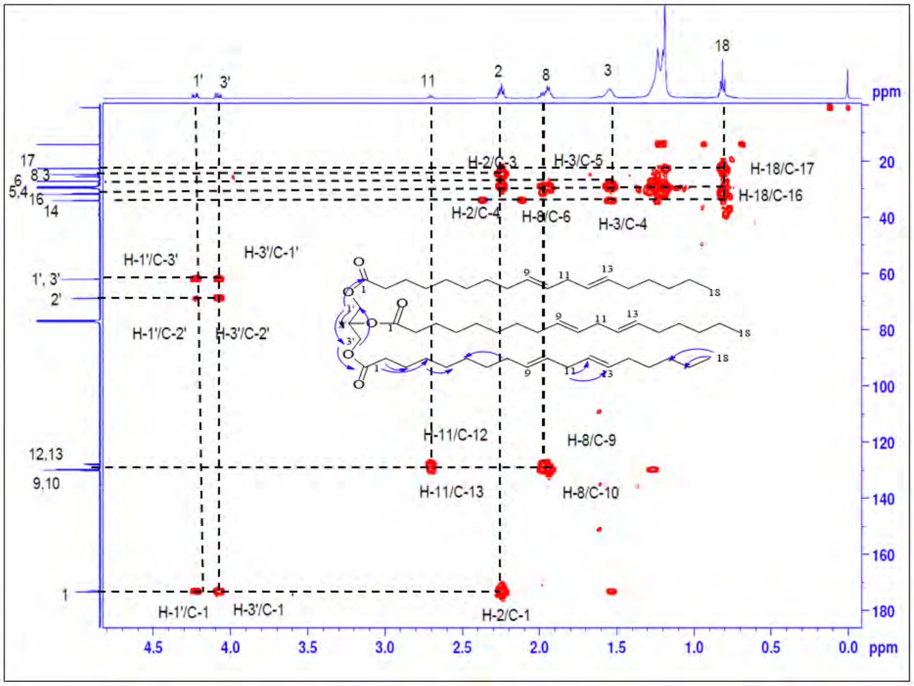

Figure S37: HMBC spectrum of **6**

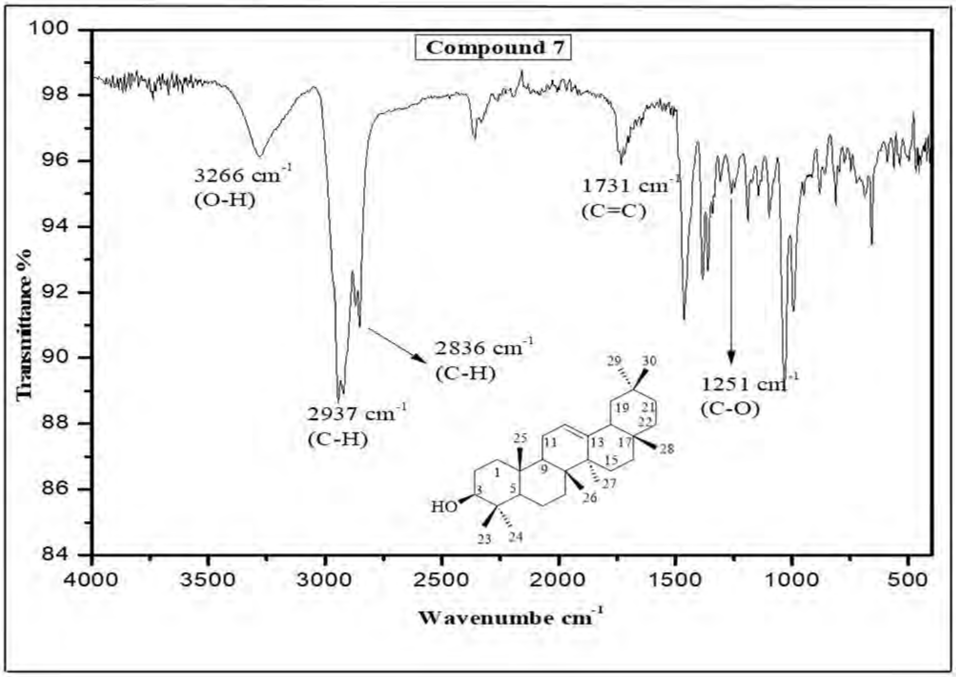

Figure S38: FT-IR spectrum of **7**

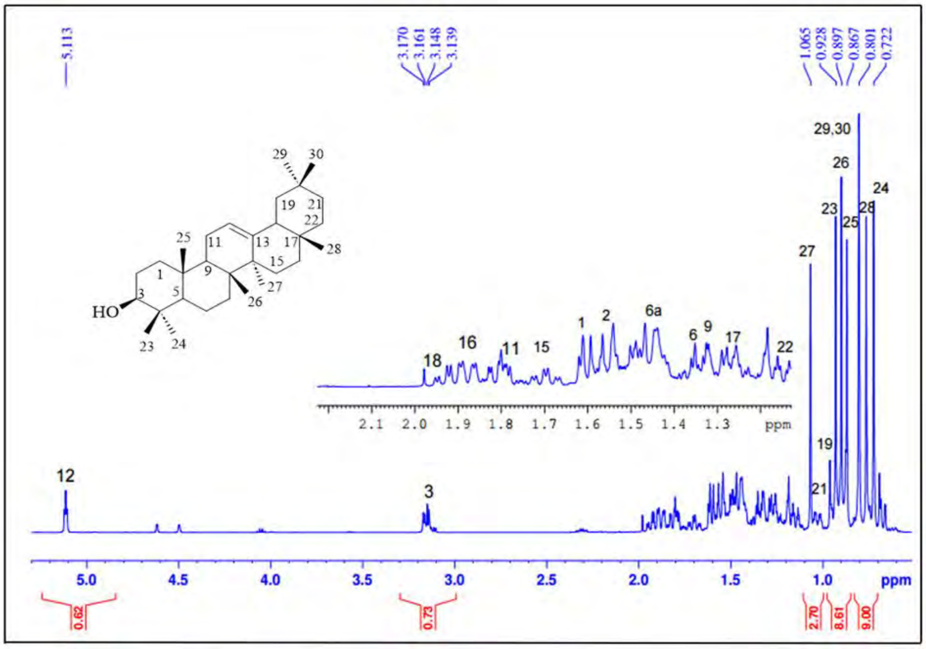

Figure S39: ^{1}H-NMR (500 MHz in $CDCl_3$) spectrum of **7**

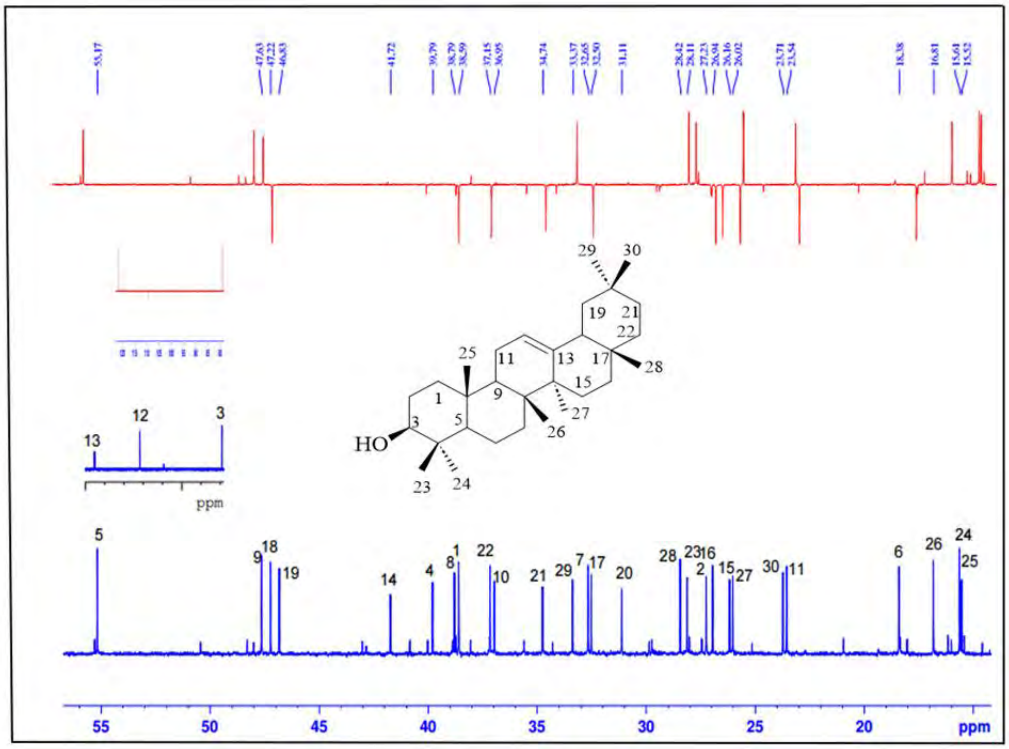

Figure S40: ^{13}C-NMR and DEPT-135 (125 MHz in $CDCl_3$) spectrum of **7**

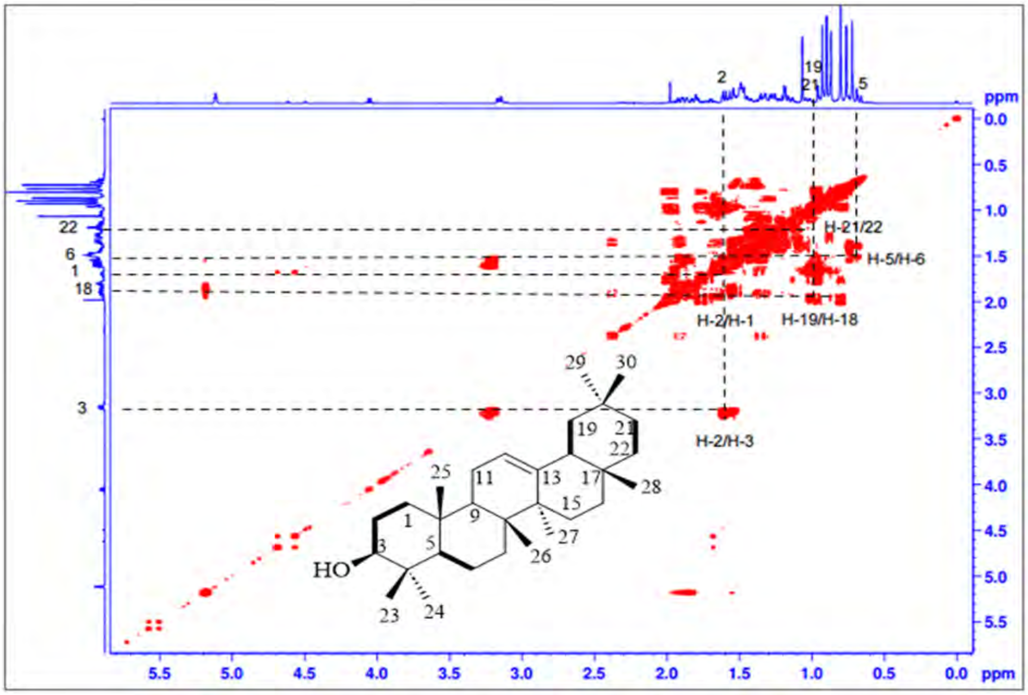

Figure S41: ^{1}H-^{1}H COSY spectrum of **7**

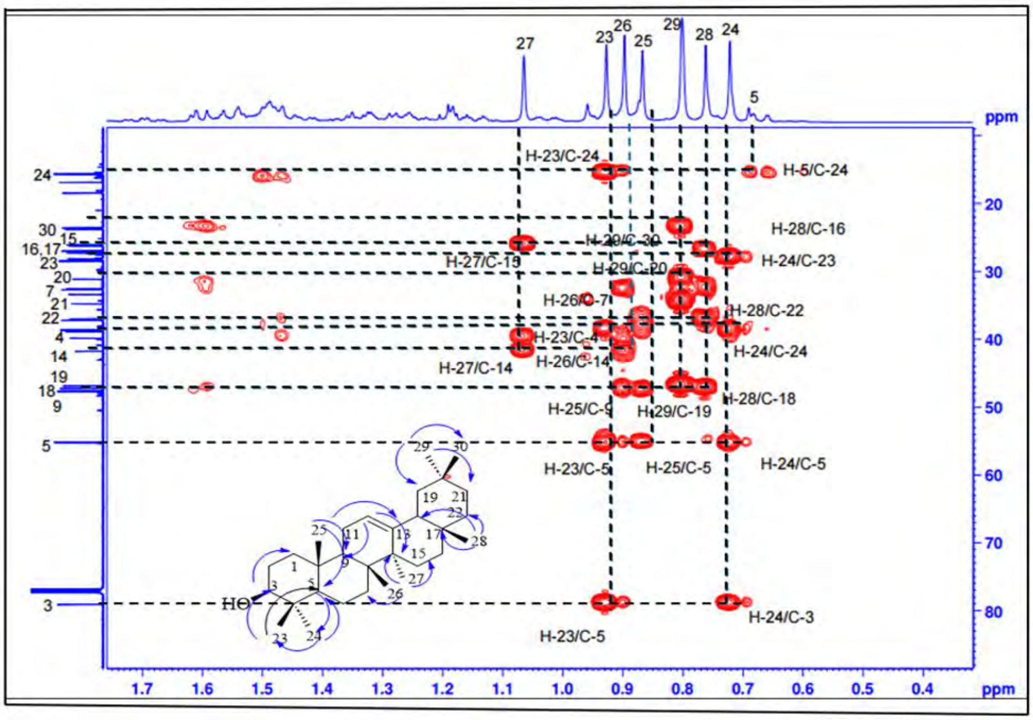

Figure S42: HMBC spectrum of **7**

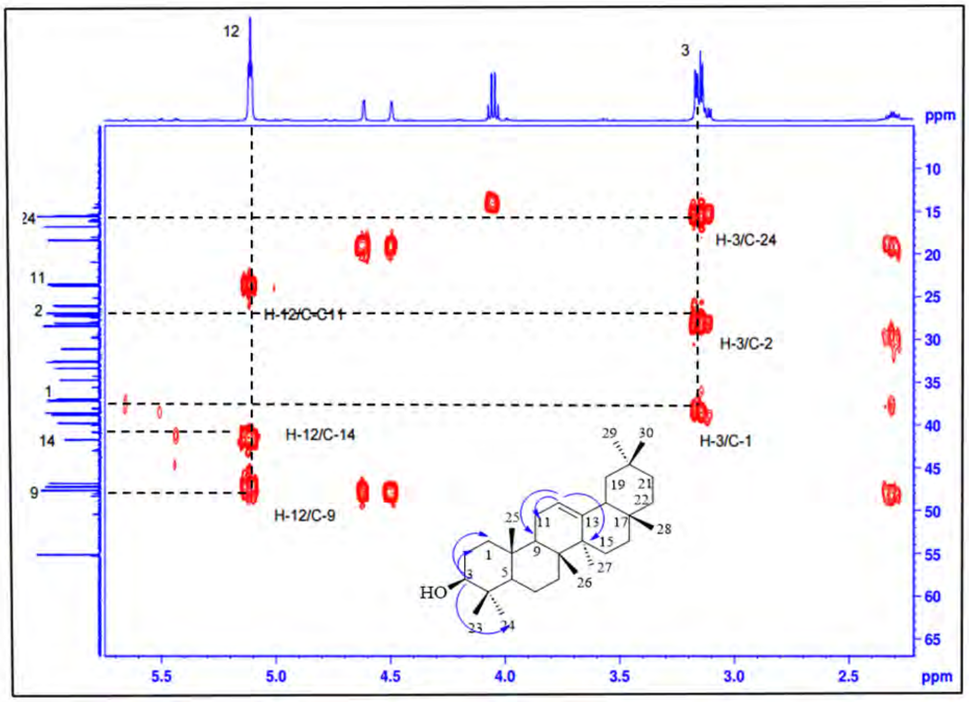

Figure S43: HMBC spectrum of **7** (expanded)

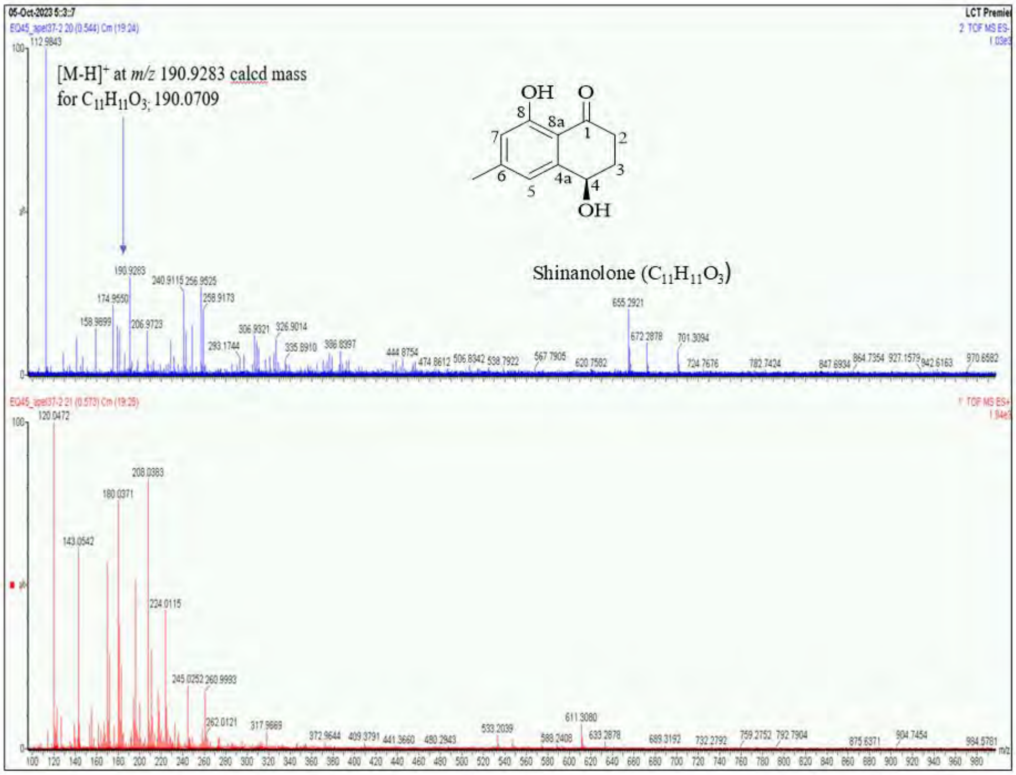

Figure S44: HRMS spectrum of **8**

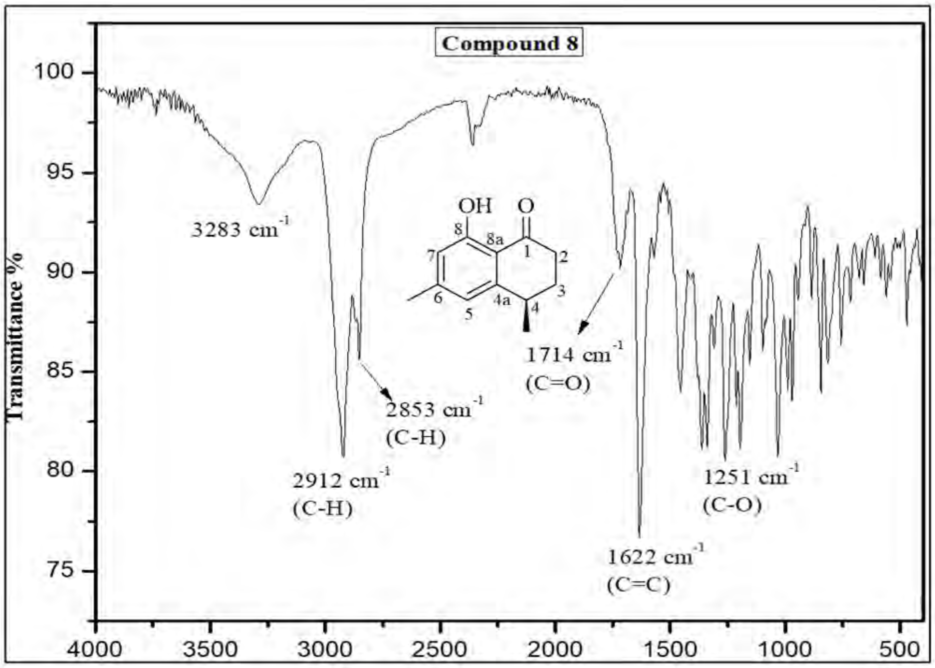

Figure S45: FT-IR spectrum of **8**

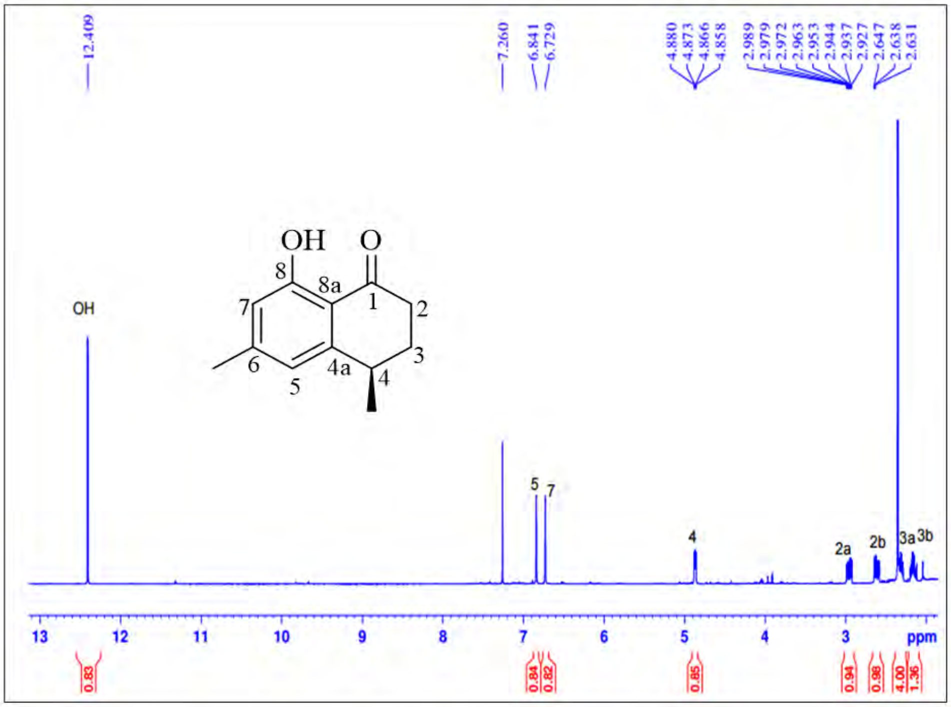

Figure S46: ^{1}H-NMR (500 MHz in $CDCl_3$) spectrum of **8**

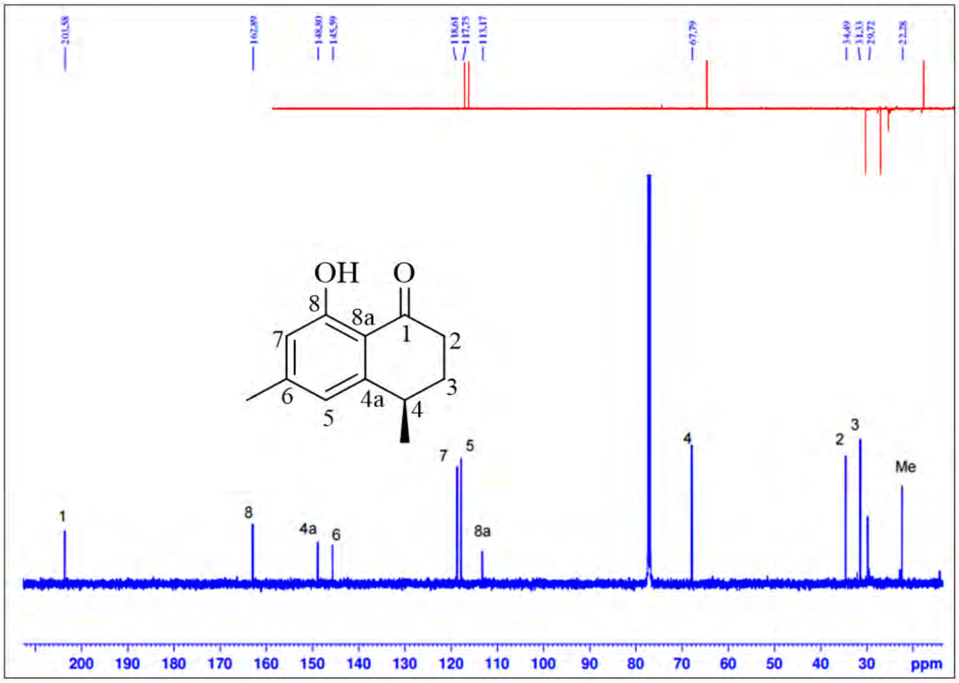

Figure S47: ^{13}C-NMR and DEPT-135 (125 MHz in $CDCl_3$) spectrum of spectrum of **8**

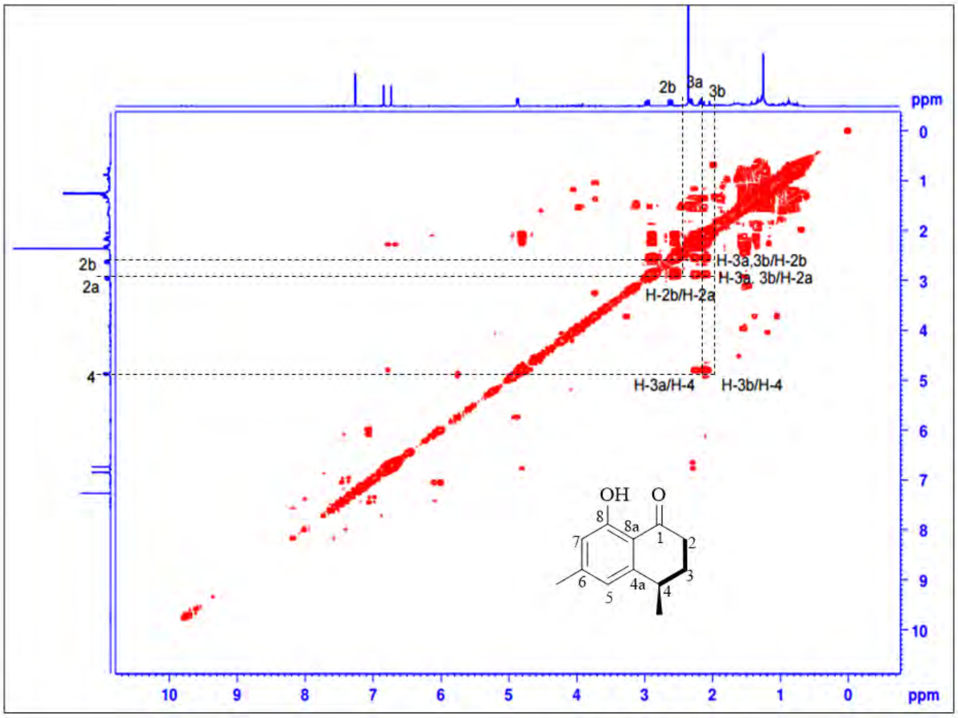

Figure S48: ^{1}H-^{1}H COSY spectrum of **8**

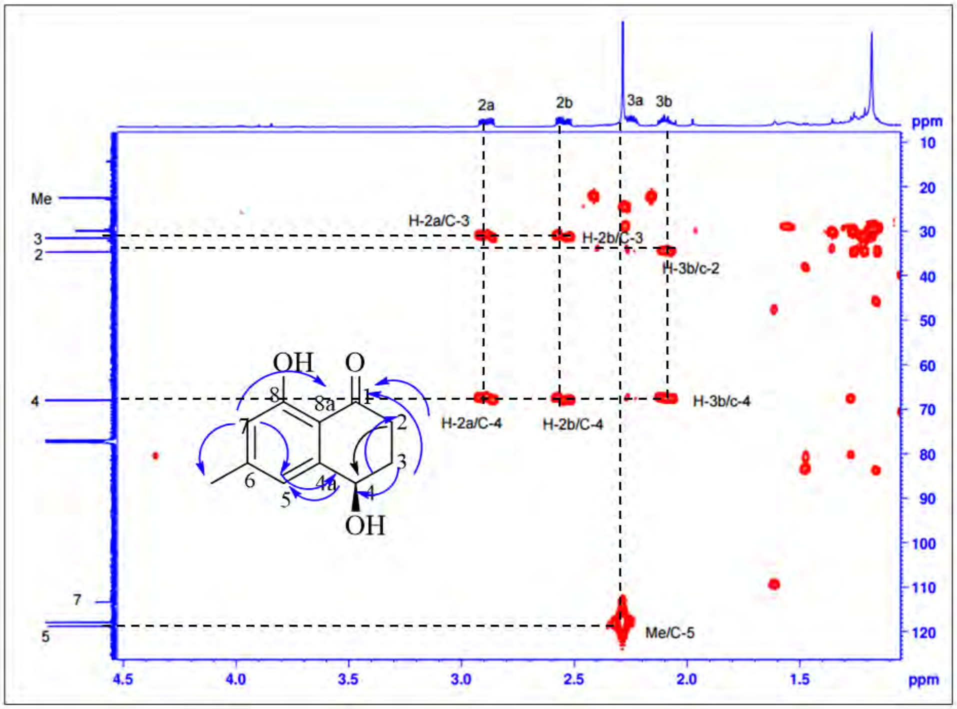

Figure S49: HMBC spectrum of **8**

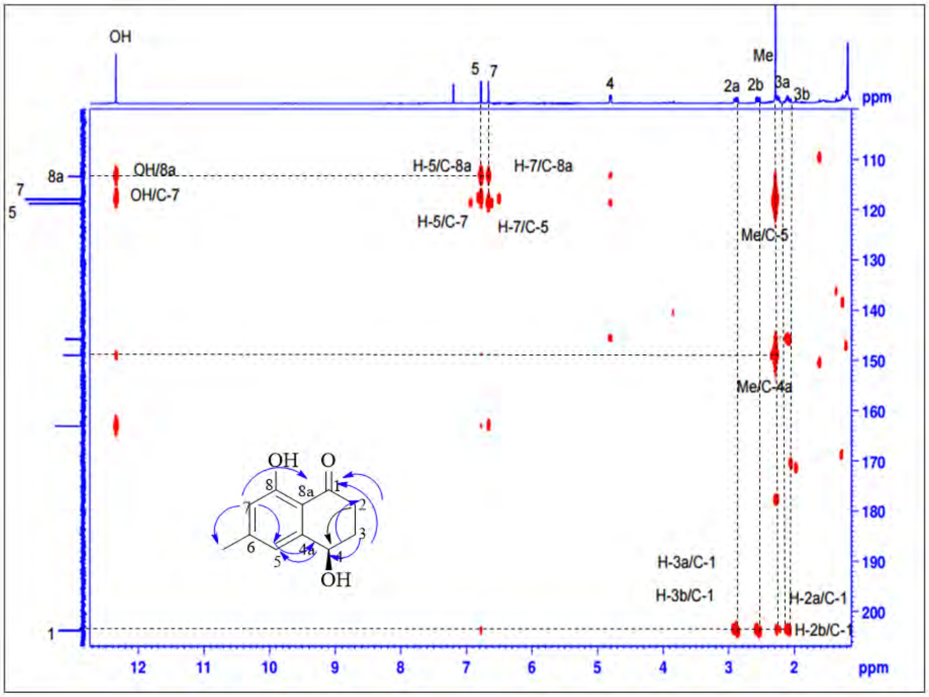

Figure S50: HMBC spectrum of **8** (Expanded)
